# DISC1 regulates lactate metabolism in astrocytes: implications for psychiatric disorders

**DOI:** 10.1038/s41398-018-0123-9

**Published:** 2018-04-12

**Authors:** Yan Jouroukhin, Yusuke Kageyama, Varvara Misheneva, Alexey Shevelkin, Shaida Andrabi, Emese Prandovszky, Robert H. Yolken, Valina L. Dawson, Ted M. Dawson, Susan Aja, Hiromi Sesaki, Mikhail V. Pletnikov

**Affiliations:** 1 Departments of Psychiatry and Behavioral Sciences, Johns Hopkins University School of Medicine, Baltimore, MD 21287 USA; 2 Departments of Pathology, Johns Hopkins University School of Medicine, Baltimore, MD 21287 USA; 3 Depart ment of Pharmacology & Toxicology, University of Alabama at Birmingham School of Medicine, Birmingham, AL 35294 USA; 4 Stanley Neurovirology Laboratory, Department of Pediatrics, Johns Hopkins University School of Medicine, Baltimore, MD 21287 USA; 5 Departments of Neurology, Johns Hopkins University School of Medicine, Baltimore, MD 21287 USA; 6 Departments of Physiology, Johns Hopkins University School of Medicine, Baltimore, MD 21287 USA; 7 Solomon H. Snyder Department of Neuroscience, Johns Hopkins University School of Medicine, Baltimore, MD 21287 USA; 8 Neuroregeneration and Stem Cell Programs, Institute for Cell Engineering, Johns Hopkins University School of Medicine, Baltimore, MD 21205 USA; 9 Departments of Pharmacology and Molecular Sciences, Johns Hopkins University School of Medicine, Baltimore, MD 21287 USA; 10 Center for Metabolic and Obesity Research, Johns Hopkins University School of Medicine, Baltimore, MD 21205 USA; 11 Departments of Cell Biology, Johns Hopkins University School of Medicine, Baltimore, MD 21287 USA; 12 Department of Neuroscience, Novosibirsk State University, Novosibirsk, Russia; 13 Department of Experimental & Clinical Neuroscience, Institute of Physiology & Basic Medicine, Novosibirsk, 630090 Russian Federation

## Abstract

Our knowledge of how genetic risk variants contribute to psychiatric disease is mainly limited to neurons. However, the mechanisms whereby the same genetic risk factors could affect the physiology of glial cells remain poorly understood. We studied the role of a psychiatric genetic risk factor, Disrupted-In-Schizophrenia-1 (DISC1), in metabolic functions of astrocytes. We evaluated the effects of knockdown of mouse endogenous DISC1 (DISC1-KD) and expression of a dominant-negative, C-terminus truncated human DISC1 (DN-DISC1) on the markers of energy metabolism, including glucose uptake and lactate production, in primary astrocytes and in mice with selective expression of DN-DISC1 in astrocytes. We also assessed the effects of lactate treatment on altered affective behaviors and impaired spatial memory in DN-DISC1 mice. Both DISC1-KD and DN-DISC1 comparably decreased mRNA and protein levels of glucose transporter 4 and glucose uptake by primary astrocytes. Decreased glucose uptake was associated with reduced oxidative phosphorylation and glycolysis as well as diminished lactate production in vitro and in vivo. No significant effects of DISC1 manipulations in astrocytes were observed on expression of the subunits of the electron transport chain complexes or mitofilin, a neuronal DISC1 partner. Lactate treatment rescued the abnormal behaviors in DN-DISC1 male and female mice. Our results suggest that DISC1 may be involved in the regulation of lactate production in astrocytes to support neuronal activity and associated behaviors. Abnormal expression of DISC1 in astrocytes and resulting abnormalities in energy supply may be responsible for aspects of mood and cognitive disorders observed in patients with major psychiatric illnesses.

## Introduction

Astrocytes are the most abundant cells in the brain, and pathological changes in astrocytes are likely to contribute to cognitive impairment and behavioral disorders^1,2^. Because of extensive tissue distribution and ramified morphology, astrocytes are in contact with neurons, glia and blood vessel cells^3,4^. Astrocytes uptake glucose and ketones to produce lactate via glycolysis or glycogenolysis, providing energy support to neurons^5^. Deficits in astrocyte energy supply can affect neuronal activity, resulting in behavioral and cognitive dysfunction^5^.

Recent progress in psychiatric genetics has advanced our knowledge of how genetic variants can affect neurodevelopment and adult brain function^6^. Unfortunately, our understanding of the underlying molecular mechanisms is limited to neurons, and little is known of the pathogenic contributions of genetic risk factors within glia, including astrocytes^7,8^.

*Disrupted-In-Schizophrenia-1* (*DISC1*) is a gene disrupted by balanced (1:11) (q42.1; q14.3) translocation, segregating in the Scottish family with several major psychiatric disorders, including schizophrenia, depression, and bipolar disorder^9^. Despite its original name, *DISC1* has not been associated with schizophrenia in the latest genome-wide association studies (GWAS)^10^ and is likely a general psychiatric risk factor that may be involved in the molecular pathogenesis of several neuropsychiatric disorders. As a genetic element of the highly penetrant ultra-rare chromosomal translocation, *DISC1* and its protein products could be used as valuable molecular tools for mechanistic studies to advance our understanding of the molecular pathobiology of several major psychiatric diseases, irrespective of their categorical classifications^11^.

Multiple reports have demonstrated that DISC1 localizes to mitochondria and is involved in mitochondria trafficking and functions, including oxidative phosphorylation, ATP production and calcium buffering^12–18^. However, all these studies were performed on neuronal models^17,19^ and whether the same partners, underlying mechanisms and/or functional consequences of altered expression of DISC1 can be observed in other brain cells remains unknown although a recent report demonstrated cell-type-specific molecular changes due to expression of DISC1 variants^20^. Thus, we sought to determine the role(s) of DISC1 in energy metabolism in astrocytes. Our findings show, for the first time, that both knockdown of endogenous DISC1 and expression of a dominant-negative, C-terminus truncated human DISC1 (DN-DISC1) produced decreased levels of the glucose transporter 4 (GLUT4), diminished uptake of glucose, and reduced production of lactate by astrocytes. The metabolic perturbations in DN-DISC1 mice were associated with altered affective behaviors and deficient memory that were rescued by systemic lactate treatment.

## Materials and methods

### General design

To study the role of DISC1 in energy metabolism in astrocytes, we employed two complementary approaches: knockdown of endogenous mouse *Disc1* (DISC1-KD) in primary astrocytes or expression of a dominant-negative, C-terminus truncated human DISC1 (DN-DISC1) in primary astrocytes or mice.

### Measurement of mitochondrial membrane potential (ΔΨ) and mitochondrial morphology

To determine the effects of DISC1-KD or DN-DISC1 on the morphology or the membrane potential of mitochondria, primary astrocytes were incubated with 100 nM of the membrane-potential-independent MitoTracker Green (MTG, M7514, Invitrogen-Molecular Probes) and 8 nM of the membrane-potential-dependent tetramethylrhodamine, ethyl ester (TMRE, T669, Invitrogen-Molecular Probes) for 30 min. Afterwards, cells were gently washed three times with PBS and incubated with live cell imaging solution (A14291DJ, Molecular Probes). Live cells were imaged using a confocal microscope (LSM 510 Meta, Carl Zeiss) with a 100× oil-immersion objective. MTG was excited with a 488-nm argon laser, and emission was recorded with a 500 to 550-nm bandpass filter. TMRE was excited with a 543-nm helium-neon laser, and emission was recorded with a 650 to 710-nm bandpass filter. Data were analyzed and are presented as the TMRE/MTG ratio. The ratio of the area occupied by swollen, spherical mitochondria with reduced ΔΨ^21^ to the area occupied by all mitochondria in a given astrocyte was calculated. At least 20 cells were evaluated for each group.

### Measurements of oxygen consumption rate (OCR)

The mitochondrial OCR was assessed in primary astrocytes using an XF24 Extracellular Flux Analyzer (Seahorse Bioscience) as described previously^22^. Briefly, approximately 100,000 primary astrocytes were plated at a density of 50 × 10^3^ per well in XF24 cell-culture microplates pre-coated with poly-d-lysine (Sigma P6407). The cultures were incubated for 24 h in growth medium. The next day, the medium was replaced with Seahorse XF medium supplemented with 10 mM glucose (Sigma, G8769), 1 mM l-glutamine (Life Technologies, 25030081) and 1 mM sodium pyruvate (Life Technologies, 11360070). OCR was analyzed in an XF24 analyzer after 45 min of incubation at 37 °C in a CO_2_-free incubator. The first OCR measurement was recorded following 11-min equilibration, a 1-min mix period, and a 1-min wait period. Oligomycin, carbonyl cyanide m-chlorophenylhydrazone, (CCCP) and rotenone were sequentially added to each well to assess basal respiration, coupling of the respiratory chain, and mitochondrial respiratory capacity, respectively.

### Measurement of glycolysis as extracellular acidification rate

Primary astrocytes were plated at a density of 50 × 10^3^ per well in XF24 cell-culture microplates for 48 h. On the day of assay, the medium was replaced with glucose-free XF24 Seahorse medium. Glycolytic flux (basal glycolysis, glycolytic capacity, and glycolytic reserve) was assessed as the extracellular acidification rate (ECAR) by sequentially adding glucose, oligomycin, and 2-deoxyglucose to the XF24 flux analyzer^23^. ECAR was measured at 37 °C with a 1-min mix, 1-min wait, and 2-min measurement protocol. Seahorse analysis was started after 45 min of incubation in a CO_2_-free incubator. The first ECAR measurement was recorded after 11-min equilibration, a 1-min mix period, and a 1-min wait period.

### Glucose uptake assay

Glucose uptake was measured in culture medium using a glucose uptake cell-based assay kit (600470, Cayman). Primary astrocytes were seeded the day before at a density of 10,000 cells per well in 96-well plates. The fluorescently labeled glucose analog 2-N-(7-nitro-benz-2-oxa-1,3-diazol-4-yl) amino)-2 deoxyglucose (2-NBDG) was used to measure glucose uptake. Primary astrocytes were incubated with 2-NBDG for 30 min and then washed three times with phosphate-buffered saline (PBS). Cell lysate was used to detect the fluorescence signal at the excitation and emission wavelengths of 485 and 535 nm, respectively, using a GloMax Discover System (Promega) luminometer in a 96-well microplate.

### Lactate measurement in primary astrocytes

Lactate was measured in medium using an l-lactate assay kit (MAK065, Sigma). Cells were seeded at a density of 20,000 cells per well in 96-well plates pre-coated with poly-d-lysine. The next day, the medium was replaced with FBS-free medium (Life Technologies, 11965092). After 1 h of incubation, the medium was collected and centrifuged at 10,000×*g* for 5 min. The secreted l-lactate concentration in the supernatant was colorimetrically determined by an absorbance measurement at 570 nm.

### DN-DISC1 mouse model

To assess the role of DISC1 in energy metabolism in vivo, we utilized an inducible dominant-negative DISC1 (DN-DISC1) model generated using the Tet-off double transgenic (Tg) system as previously described^24^. Briefly, one Tg line (regulatory line) expresses the tetracycline transactivator (tTA) protein that binds to TetO sequences present on another Tg line (responder line or tetracycline response element (TRE) line). In our model, mice of the regulatory line are single transgenic GFAP-tTA mice (line 67, a kind gift from Dr. Brian Popko, University of Chicago) that express tTA predominantly in astrocytes^25^. Hemizygous GFAP-tTA mice were crossed with homozygous TRE mice (line 1001) that express C-terminus truncated human protein. The protein product acts as a dominant-negative factor (DN-DISC1) by binding to and decreasing the expression of endogenous mouse DISC1^24^. Mice of both lines were back-crossed to C57BL/6j background for more than 15 generations. This mating protocol produces litters with ~50% control mice (TRE-DN-DISC1 mice) that do not express DN-DISC1 as they lack tTA and ~50% DN-DISC1 mice that are double Tg mice and express DDN-DISC1 predominantly in astrocytes. Tail tissue samples were used for genotyping as previously described^24^. Developing mice were housed with their dams until postnatal days (P) 21–23, with food and water provided ad libitum. All procedures were approved by the JHU Animal Care and Use Committee.

### Indirect calorimetry and energy balance measurements

To determine whether DN-DISC1 expression in astrocytes affects whole-body metabolism, male DN-DISC1 (*n* = 10) and control (*n* = 13) mice were monitored individually in an open-circuit indirect calorimeter with additional features to measure ad libitum food intake and physical activity (Comprehensive Lab Animal Monitoring System, CLAMS, Columbus Instruments, Columbus OH). Mice were monitored for four consecutive days, the first three of which involved confirming behavioral and metabolic adaptation. Rates of O_2_ consumption (VO_2_) and CO_2_ production (VCO_2_) were measured every 24 min per mouse and input into software (Oxymax V.4.93) to calculate respiratory exchange ratios (RER = VCO2/VO2) and rates of energy expenditure (EE)^26^. Oxymax calorimetry data outputs were on a per-kg body weight basis, then renormalized to estimate per-kg-lean mass, utilizing body composition data from the mice measured in an EchoMRI-100 immediately prior to CLAMS. Calorimetry data, as well as food intake and physical activity data, are presented in 4-h bins of averaged data per mouse, averaged for each group.

### Lactate assay in brain tissue and blood

We also measured lactate levels in the hippocampus and blood serum of control and DN-DISC1 2-month-old mice. Mice were sacrificed, and their hippocampi and blood samples were collected and processed for lactate measurements according to the manufacturer’s method (Lactate Assay Kit II, MAK065, Sigma-Aldrich).

### Behavioral tests

Behavioral tests were performed on 2-month-old control and DN-DISC1 male and female mice. The elevated plus maze (EPM), the forced swim test (FST) and trace fear conditioning (TFC) were used to assess the behavioral effects of preferential l-lactate administration. EPM and FST were described in detail in our previous publications^24,27–29^. TFC was a 3-day test consisting of a habituation day, training day, and test day. Mice were habituated to the shock box (Coulbourn, Holliston, MA) for 10 min. The following day, mice were placed in the shock box, and a 20-s white noise tone was delivered. Twenty seconds following the termination of the tone, a scrambled 2-s 0.5-mA shock was delivered. This tone-shock pairing was repeated three times. On the third day, mice were placed in the shock box for 3 min to measure freezing in response to the context. Following the exposure to the shock box, mice were placed in a different box (a new context not associated with the shock), and the 20-s white noise tone was delivered, during which freezing behavior in response to the tone was measured.

### Lactate treatments

Mice received single intraperitoneal (ip) injections of vehicle or l-lactate (1 mg/kg) or d-lactate (1 mg/kg) 1 h prior to the tests.

### Statistical analyses

Normality and equal variance tests were performed before the following statistical tests were applied to data. Data are expressed as means ± standard error of the mean (SEM). No statistical methods were used to estimate sample sizes. We determined animal numbers by considering our previous studies on the same mouse model and behavioral tests^25^. All experiments were conducted in triplicate (cell culture and biochemical studies) or with three cohorts of animals (behavioral studies). Mice were randomly allocated to different treatment groups. No animals or data points were excluded from analyses. Although data collection and analysis were not performed with blinding to the experimental conditions, the same analysis and comparison criteria were applied to all cell samples or mice.

OCR and ECAR data were analyzed by two-way repeated analyses of variance (ANOVA). The behavioral and lactate treatment data were analyzed using ANOVA or Student’s *t*-test when appropriate. In vitro and biochemical data were analyzed by two-tailed Student’s *t*-tests. We used the *F*-test to evaluate the group variance prior to each *t*-test. Bonferroni correction was applied to multiple comparisons; the significance level was set to *p* < 0.05.

All raw data are available to the scientific community upon reasonable request.

## Results

### No morphological changes in astrocyte mitochondria

**Fig. 1:**
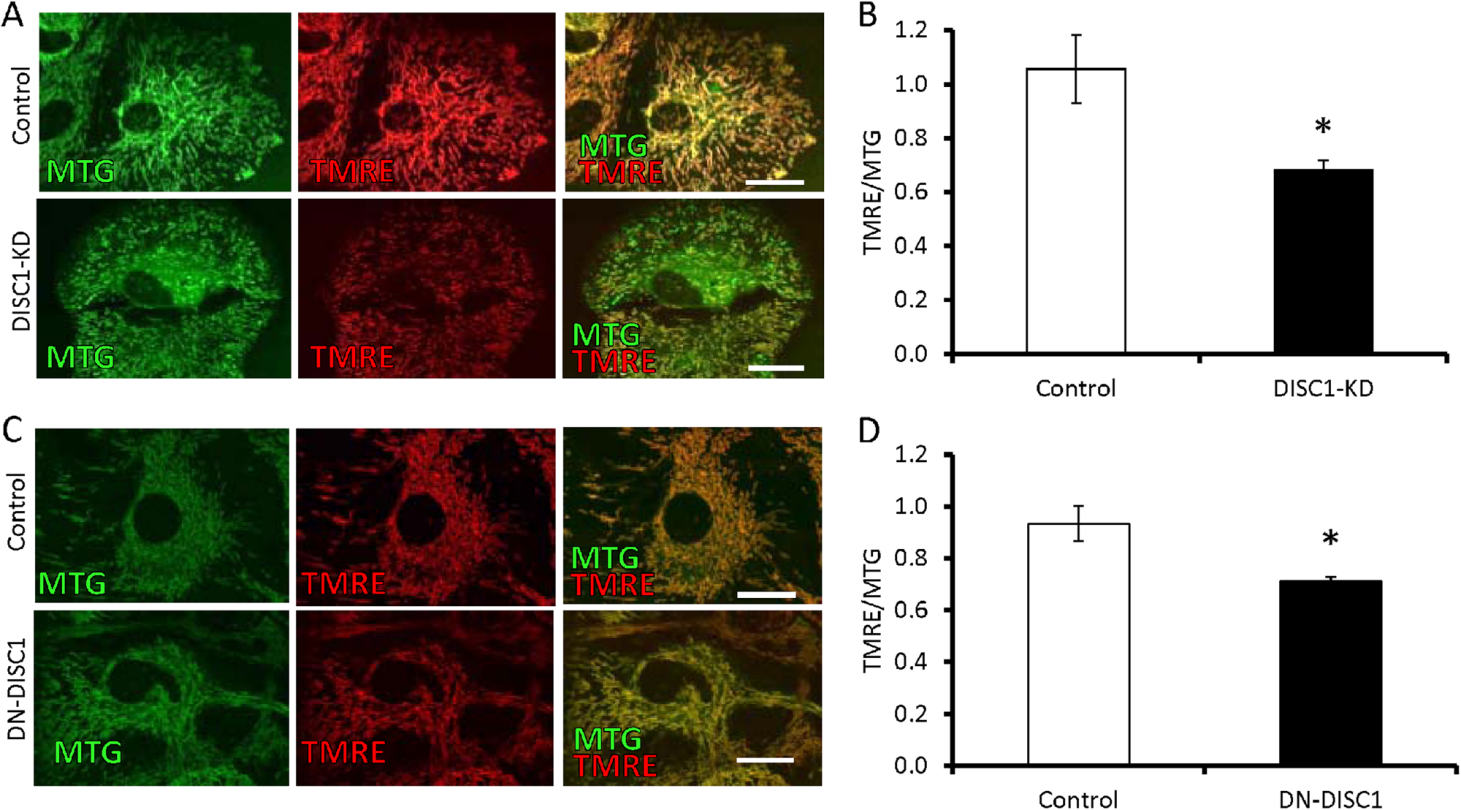
Decreased mitochondrial membrane potential in primary astrocytes. Both DISC1-KD and DN-DISC1 expression in primary astrocytes led to a decreased mitochondrial membrane potential. Primary astrocytes were incubated for 30 min with the membrane-potential-sensitive dye TMRE (red) and mitochondrial membrane dye MRG (green). The membrane potential was assessed as the TMRE/MTG ratio; 3 independent primary astrocyte cultures were used for each condition; Scale bars—50 μm **DISC1-KD**: **a** Representative images of control (upper row) and DISC1-KD primary astrocytes (bottom row); **b** quantitative analysis of the membrane potential of control and DISC1 KD astrocytes; *n* = 3 independent cultures; each culture was measured in triplicate (at least 30 cells per culture were evaluated); *p < 0.05, two-tailed Student’s *t*-test, *t* = 3.1 **DN-DISC1**: **c** Representative images of control (upper row) and DN-DISC1 primary astrocytes (bottom row); **d** quantitative analysis of the membrane potential of control and DN-DISC1 astrocytes; *n* = 3 independent cultures; each culture was measured in triplicate (at least 30 cells per culture were evaluated); *p < 0.05, two-tailed Student’s *t*-test, *t* = 5.5; data are presented as means ± SEM

We studied the role of DISC1 in energy metabolism in astrocytes using two approaches: DISC1 knockdown (DISC1-KD) (Supplemental Figure S1) and astrocyte selective expression of a dominant-negative, C-terminus truncated human DISC1 (DN-DISC1). Although both endogenous and DN-DISC1 co-localized to mitochondria in primary astrocytes (Supplemental Figure S2-S3), neither DISC1-KD nor DN-DISC1 had any significant effects on the number or shape of mitochondria, including the percentage of abnormal round swollen mitochondria in astrocytes (Supplemental Figure S4). In addition, neither treatment significantly changed the expression of the electron transport chain subunits (Supplemental Fig. 5), the protein import translocase Tim23 (Supplemental Figure 6), or mitofilin, a neuronal DISC1 partner in mitochondria^30^ (Supplemental Figure 7). However, both DISC1-KD and DN-DISC1 significantly decreased the mitochondrial membrane potential, as assessed by TMRE staining (Fig. 1), suggesting abnormal functioning of astrocyte mitochondria produced by altered expression of DISC1.

### Abnormal energy metabolism in astrocytes

**Fig. 2:**
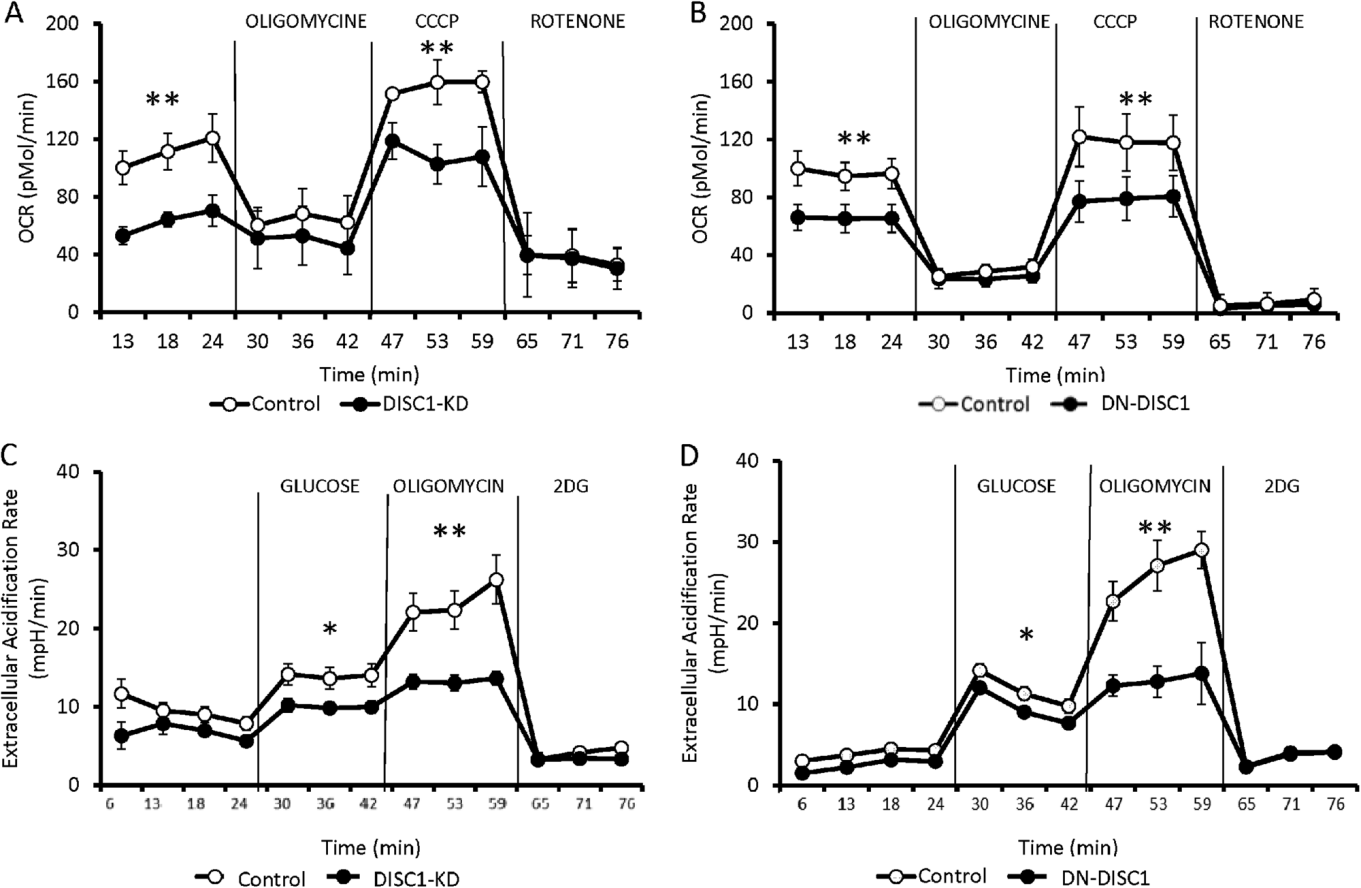
Metabolic abnormalities in primary astrocytes. Both DISC1-KD and DN-DISC1 inhibited respiration and glycolysis in primary astrocytes. The oxygen consumption rate (OCR) and extracellular acidification rate (ECAR) were measured by Seahorse analysis in primary astrocytes. OCR was measured using the Seahorse XF Cell Mito Stress test after injection of the mitochondrial ETC inhibitors oligomycin (an inhibitor of complex V), CCCP (which uncouples the proton gradient), and rotenone (an inhibitor of complex I). ECAR was measured using the Seahorse XF Glycolysis Stress test after injection of glucose, oligomycin and 2-deoxyglucose (2DG). **DISC1-KD**: **a** Representative OCR for control (open circle) and DISC1-KD (solid circle) primary astrocytes; *n* = 5 independent cultures; each culture was measured in duplicate, and each experiment was repeated at least three times. Two-way repeated measures ANOVA revealed a significant effect of KD, F(1, 23) = 101,69, *P* < 0.01; a post hoc Bonferroni test showed that basal respiration (*P* < 0.05) and the maximal respiration rate measured after CCCP injection (*P* < 0.05) were significantly lower in DISC1-KD astrocytes than in control astrocytes; * *p* < 0.05. **b** ECAR for control (open circle) and DISC1-KD (solid circle) primary astrocytes; *n* = 5 independent cultures; each culture was measured in duplicate, and each experiment was repeated at least three times. Two-way repeated measures ANOVA revealed a significant effect of KD, F(1, 23) = 175.7, *p* < 0.01; a post hoc Bonferroni test showed that basal glycolysis (*P* < 0.05) and glycolytic capacity measured after oligomycin injection (*P* < 0.01) were significantly lower in DISC1-KD astrocytes than in control astrocytes; * *p* < 0.05, **p < 0.01. **DN-DISC1**: **a** Representative OCR for control (open circle) and DN-DISC1 (solid circle) primary astrocytes; *n* = 5 independent cultures; each culture was measured in duplicate, and each experiment was repeated at least three times. Two-way repeated measures ANOVA showed a significant effect of DN-DISC1, F(1, 23) = 21.37, *P* < 0.05; a post hoc Bonferroni test showed that basal respiration (*P* < 0.05) and the maximal respiration rate measured after CCCP injection (*P* < 0.05) were significantly lower in DN-DISC1 astrocytes than in control astrocytes; *p < 0.05. **b** ECAR for control (open circle) and DISC1-KD (solid circle) primary astrocytes; *n* = 5 independent cultures; each culture was measured in duplicate, and each experiment was repeated at least three times. Two-way repeated measures ANOVA showed a significant effect of DN-DISC1, F(1, 23) = 136.11, *P* < 0.001; a post hoc Bonferroni tests showed that basal glycolysis (*P* < 0.05) and glycolytic capacity measured after oligomycin injection (*P* < 0.01) were significantly lower in DN-DISC1 astrocytes than in control astrocytes; *p < 0.05, **p < 0.01; data are presented as means ± SEM

To provide a more comprehensive evaluation of the potential role of DISC1 in the energy metabolism in astrocytes, we examined the effects of DISC1-KD and DN-DISC1 on oxidative phosphorylation and oxygen consumption using the Seahorse Mito stress test. Compared with control astrocytes, DISC1-KD or DN-DISC1 significantly decreased basal respiration and carbonyl cyanide m-chlorophenylhydrazone (CCCP)-induced maximal respiration in primary astrocytes (Fig. 2a, b). Somewhat unexpectedly, we also observed a significantly diminished extracellular acidification rate (ECAR) in DISC1-KD and DN-DISC1 primary astrocytes, suggesting declined glycolytic production of lactate (Fig. 2c, d). Prior reports have demonstrated that the oxygen consumption rate and glycolysis rate are two inter-dependent factors that are influenced by oxygen and glucose availability (i.e., the Pasteur and Crabtree effects)^31^. Our results suggest that altered levels of DISC1 might disturb glucose consumption, which in turn could lead to a simultaneous reduction of these two inter-dependent energy pathways in astrocytes. Thus, we hypothesized that chronically reduced uptake of glucose, the key energy substrate, might be responsible for the concurrent decrease in oxidative phosphorylation and glycolysis in DISC1 KD and DN-DISC1 astrocytes.

### Diminished glucose uptake and lactate production

**Fig. 3:**
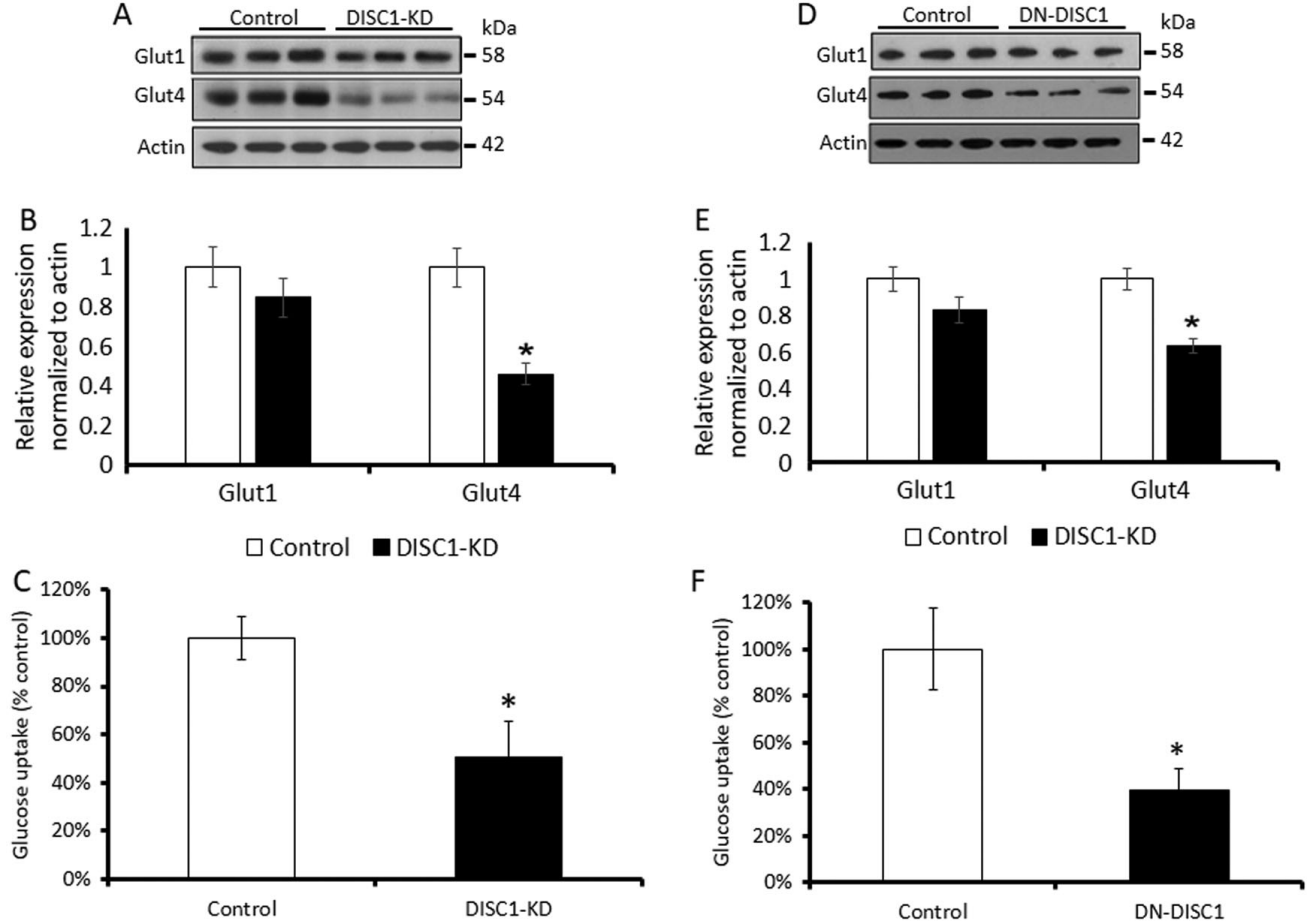
Decreased expression of GLUT4 and glucose uptake by primary astrocytes. Expression of the glucose transporters and glucose uptake were measured in primary astrocytes. Both DISC1-KD and DN-DISC1 decreased expression of GLUT4 and glucose uptake in primary astrocytes. **DISC1-KD**: **a** Representative Western blotting images of expression of the glucose transporters by control and DISC1-KD primary astrocytes; **b** quantitative analysis of the expression of the transporters by control (open bars) and DISC1-KD (solid bars) astrocytes; *n* = 3 independent cultures; each culture was measured in triplicate; *p < 0.05, two-tailed Student’s *t*-test; *t* = −4.808 for GLUT4; **c** quantitative analysis of glucose uptake by control (open bars) and DISC1-KD (solid bars) astrocytes; *n* = 4 independent cultures; each culture was measured in triplicate; *p < 0.05, two-tailed Student’s *t*-test, *t* = 3.254. **DN-DISC1**: **d** Representative Western blotting images of expression of the glucose transporters by control (open bars) and DN-DISC1 (solid bars) primary astrocytes; **e** quantitative analysis of expression of the glucose transporters by control (open bars) and DN-DISC1 (solid bars) primary astrocytes; *n* = 3 independent cultures; each culture was measured in triplicate; *p < 0.05, two-tailed Student’s *t*-test; *t* = −4.673 for GLUT4; **f** quantitative analysis of glucose uptake by control (open bars) and DN-DISC1 (solid bars) astrocytes; *n* = 4 independent cultures; each culture was measured in triplicate; *p < 0.05, two-tailed Student’s *t*-test, *t* = 3.049; data are presented as means ± SEM

To address this hypothesis, we first measured mRNA expression for the glucose transporters GLUT1 and GLUT4 expressed on astrocytes^32^. We found significantly decreased levels of mRNAs for GLUT4 and significantly increased levels of mRNA for GLUT1 (Supplemental Table 2). We also found significantly reduced protein levels of GLUT4 but not GLUT1 (Fig. 3a–d). Finally, we observed significantly lower glucose uptake by KD-DISC1 or DN-DISC1 astrocytes compared with the uptake observed for control astrocytes (Fig. 3e, f).

**Fig. 4:**
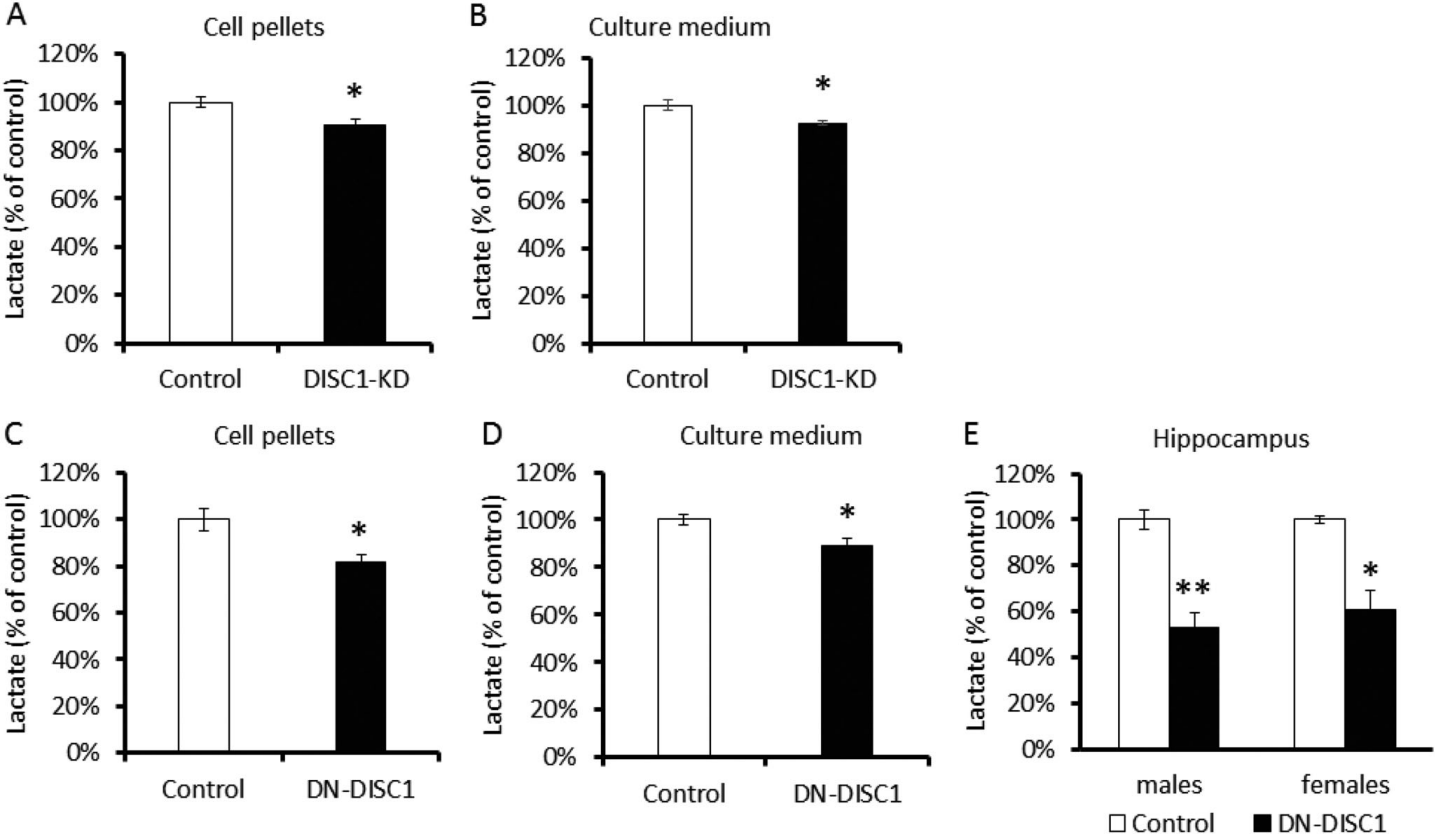
Reduced lactate production in vitro and in vivo. Lactate level was measured in primary astrocytes (cell pellets and medium) and hippocampal tissue samples using a commercial ELISA kit (MAK065, Sigma). Both DISC1-KD and DN-DISC1 decreased lactate production in primary astrocytes and the hippocampus. **DISC1-KD**: Quantitative analyses of lactate levels in cell pellets (**a**) and culture medium (**b**) in control (open bars) and DISC1-KD (solid bars) astrocytes; *n* = 4–6 independent cultures per group; each culture was measured in triplicate; two-tailed Student’s *t*-test, *t* = 2.597 for lactate measured in cell pellets, and *t* = 2.991 for lactate measured in culture medium; *p < 0.05. **DN-DISC1**: Quantitative analyses of lactate levels in cell pellets (**c**), culture medium (**d**) and the hippocampus (**e**) in control (open bars) and DN-DISC1 (solid bars) astrocytes of male and female mice; *n* = 4–6 independent cultures per primary astrocyte group; each culture was measured in triplicate; two-tailed Student’s *t*-test, *t* = 3.193 for lactate measured in cell pellets, and *t* = 2.826 for lactate measured in culture medium; *n* = 3 mice in each group measured in triplicate; *t* = 5.931 for male group, and *t* = 6.543 for female group; two-tailed Student’s *t*-test; *p < 0.05, and **p < 0.01; data are presented as means ± SEM

As lactate is a leading energy metabolite produced by astrocytes from glucose to support energy demands from neurons during their activity^33–35^, we decided to focus on lactate metabolism to determine the pathophysiological consequences of decreased glucose uptake in the brain. We assessed lactate levels in primary astrocytes and in brain tissues. Compared with control astrocytes, DISC1-KD and DN-DISC1 significantly decreased lactate levels in cell pellets and culture medium (Fig. 4a–d). However, no significant effects were observed on expression of the astrocyte-specific monocarboxylate transporter 4 (MCT4), which is responsible for lactate secretion, suggesting that decreased levels of lactate in culture medium were likely due to diminished production of lactate (Supplemental Table 2). We also observed decreased expression of the major enzymes involved in the regulation of lactate metabolism in astrocytes (Supplemental Table 2).

Consistent with our in vitro data, we detected significantly lower levels of lactate in the hippocampus of DN-DISC1 mice (Fig. 4e). Notably, we observed no significant group-dependent changes in lactate levels in blood serum (Supplemental Figure 8), suggesting that the diminished concentration of lactate in the hippocampus of DN-DISC1 mice likely results from a reduced production of lactate by brain astrocytes rather than from global changes in lactate metabolism^35^. These results are corroborated by the absence of significant genotype-related differences in the whole-body energy balance measures, as assessed by the CLAMS experiments (Supplemental Figure 9–11).

### Lactate treatment rescues behavioral alterations in DN-DISC1 mice

**Fig. 5:**
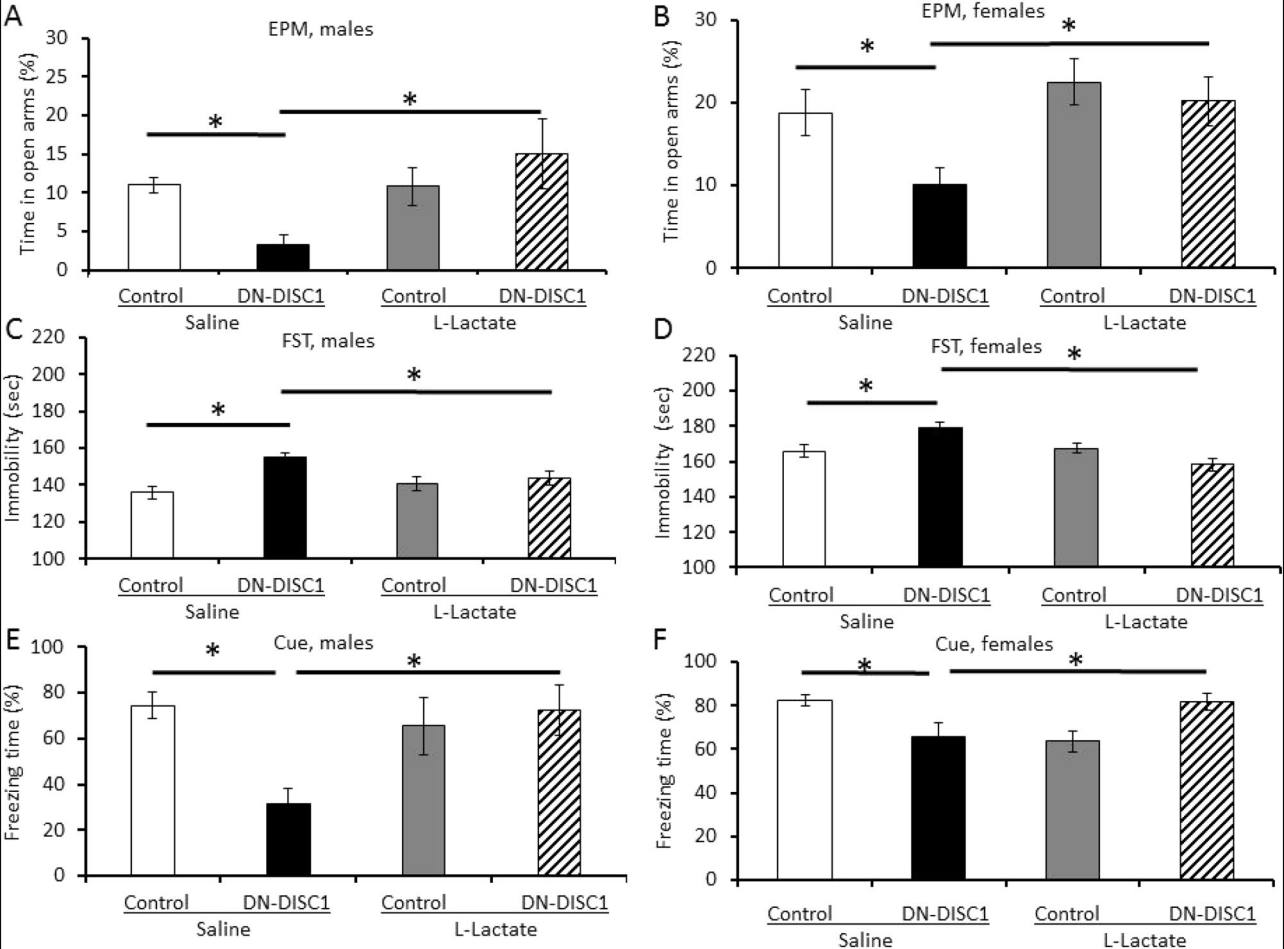
l-lactate administration rescues the behavioral changes in mice. Control and DN-DISC1 male and female 2-month-old mice received a single injection of L-lactate at a dose of 1 mg/kg (IP) 60 min before behavioral testing. Lactate treatment rescued the behavioral abnormalities in DN-DISC1 mice. **EPM**: Lactate administration significantly increased time spent in open arms of the EPM in DN-DISC1 male (**a**) and female (**b**) mice; *n* = 4–6 male and 6–11 female mice in each group. For male mice, two-way ANOVA showed a significant genotype x treatment interaction, F(1, 22) = 5.197, *p* = 0.036. A post hoc Bonferroni test showed that compared with saline-treated control mice, saline-treated DN-DISC1 mice spent significantly less time in open arms of the EPM (*p* = 0.026), and compared with saline-treated DN-DISC1 mice, lactate-treated DN-DISC1 mice spent significantly more time in open arms of the EPM (*p* = 0.022). For female mice, two-way ANOVA showed a significant effect of treatment, F(1, 29) = 5.532, *p* = 0.026; a post hoc Bonferroni *t*-test showed that compared with saline-treated control mice, saline-treated DN-DISC1 mice spent significantly less time in open arms of the EPM (*p* = 0.047), and compared with saline-treated DN-DISC1 mice, lactate-treated DN-DISC1 mice spent significantly more time in open arms of the EPM (*p* = 0.007). **FST**: Lactate administration significantly decreased immobility in FST in DN-DISC1 male (**c**) and female (**d**) mice; *n* = 6 male and *n* = 7–11 female mice in each group. *For male mice*, two-way ANOVA showed a significant effect of genotype, F(1, 22) = 11.00, *p* = 0.003, and a significant genotype by treatment interaction, F(1, 22) = 5.64, *p* = 0.027. A post hoc Bonferroni test showed that compared with saline-treated control mice, saline-treated DN-DISC1 mice exhibited significantly greater immobility (*p* < 0.001), and compared with saline-treated DN-DISC1 mice, lactate-treated DN-DISC1 mice showed significantly less immobility in the FST (*p* = 0.032). *For female mice*, two-way ANOVA showed a significant effect of treatment, F(1, 28) = 6.33, *p* = 0.018, and a genotype by treatment interaction, F(1, 28) = 8.61, *p* = 0.007. A post hoc Bonferroni test showed that compared with saline-treated control mice, saline-treated DN-DISC1 mice exhibited significantly greater immobility (*p* = 0.041), and compared with saline-treated DN-DISC1 mice, lactate-treated DN-DISC1 mice showed significantly less immobility in the FST (*p* = 0.001). **TFC**: Lactate administration significantly increased cue-dependent freezing in TFC in DN-DISC1 male (**e**) and female (**f**) mice; *n* = 4–6 male and 7–15 female mice in each group. For male mice, two-way ANOVA showed a significant genotype by treatment interaction, F(1, 17) = 6.37, *p* = 0.022. A post hoc Bonferroni test showed that compared with saline-treated control mice, saline-treated DN-DISC1 exhibited significantly less cue-dependent freezing (*p* < 0.006), and compared with saline-treated DN-DISC1 mice, lactate-treated DN-DISC1 mice showed significantly more cue-dependent freezing (*p* = 0.009). For female mice, two-way ANOVA showed a significant genotype by treatment interaction, F(1, 43) = 10.73, *p* = 0.002. A post hoc Bonferroni test showed that compared with saline-treated control mice, saline-treated DN-DISC1 mice exhibited significantly less cue-dependent freezing (*p* = 0.044), and compared with saline-treated DN-DISC1 mice, lactate-treated DN-DISC1 mice showed significantly more cue-dependent freezing (*p* = 0.016); data are presented as means ± SEM

Prior reports have demonstrated the role of lactate produced by astrocytes in cognitive function and anxiety- and depression-like behaviors in laboratory animals^36,37^. Thus, we sought to evaluate the effects of lactate treatments on the abnormal behaviors reported by our group for transgenic mice with astrocyte-restricted expression of DN-DISC1^38^. We found that a single acute injection of lactate (1 mg/kg, ip) significantly decreased anxiety-like behavior in EPM (Fig. 5a, b) and immobility in FST (Fig. 5c, d) and increased cue-dependent freezing in TFC in DN-DISC1 mice. Notably, no significant effects of lactate treatment were observed on locomotor activity in EPM or context-dependent freezing in TFC (Supplemental Figure 12). Furthermore, the enantiomer d-lactate produced no significant effects on the baseline genotype-related differences in these three tests (Supplemental Figures 13–14). Collectively, our pharmacology data indicate that acute systemic administration of l-lactate can rescue the behavioral abnormalities in DN-DISC1 mice.

## Discussion

Our findings demonstrate, for the first time, that DISC1 is involved in the regulation of energy metabolism in astrocytes. Both DISC1 knockdown and expression of a dominant-negative DISC1 (DN-DISC1) decreased expression of the glucose transporter 4 (GLUT4), leading to diminished uptake of glucose and decreased production of lactate. These metabolic changes in mice with astrocyte selective expression of DN-DISC1 could contribute to abnormal affective behaviors and deficient spatial memory reversible by systemic lactate treatment.

The present findings significantly expand our knowledge of the roles of DISC1 in different brain cell types with respect to energy metabolism. Prior studies have consistently implicated DISC1 in mitochondrial functions, including mitochondria trafficking, ATP production and Ca^2+^ buffering^39–41^. However, all reports have focused on neuronal DISC1. As various genetic risk factors could play variable, cell-type-specific roles in the pathogenesis of psychiatric disorders^20,42^, it is important that we elucidate the contribution of genetic variants expressed in different brain cells. In this context, our work provides new insights into the possible role of DISC1 in energy metabolism in astrocytes. Our findings suggest that altered DISC1 expression in astrocytes affects glucose uptake, which leads to diminished lactate production, one of the major energy supply processes in astrocytes^35,43,44^. Because both DISC1-KD and DN-DISC1 approaches to manipulating DISC1 expression in astrocytes produced comparable outcomes, we believe that potential off-target effects of either manipulation are less likely, suggesting that the present study points to the critical role of DISC1 in energy metabolism in astrocytes.

Although the current study does not directly address the mechanisms of how DISC1 is involved in glucose uptake regulation, one could propose that, as a scaffolding protein^45^, DISC1 is involved in trafficking and/or membrane recycling of glucose transporters^46^. Astrocytes express two glucose transporters, GLUT1 and GLUT4. Compared with that of GLUT1, expression of GLUT4 in astrocytes is 20 times lower^47^. GLUT4 is an insulin-sensitive glucose transporter that is translocated to the cell membrane when insulin activates PI3K/Akt signaling^48,49^. GLUT4 is highly expressed in the hippocampus, olfactory bulbs and cerebellum^50,51^. Although the role of insulin signaling in the brain is poorly understood, previous studies have suggested that hippocampal GLUT4 could play a key role in learning and memory^52^. Notably, we found that astrocyte-specific expression of DN-DISC1 decreased levels of GLUT4 in the hippocampus and impaired performance in trace fear conditioning.

Unlike GLUT4 and despite decreased glucose uptake, we found no significant changes in protein levels of GLUT1. However, given the major role of GLUT1 in glucose uptake in astrocytes, the reason for the inconsistency between unaltered GLUT1 levels and reduced glucose uptake remains unclear. It is possible that impaired trafficking of GLUT1 in DISC1-KD and DN-DISC1 astrocytes could lead to comparable levels of GLUT1, as assessed by Western blotting and reduced glucose uptake. For example, DISC1-KD has been reported to decrease activation of PI3K^53^, which regulates intracellular transport of GLUT1 to the plasma membrane^54–59^. In addition, GLUT1 intracellular transport could be impaired due to abnormal DISC1-mediated activation of RHOA/ROCK1^60^ and/or RAS^57,61,62^. Thus, it is tempting to speculate that DISC1-KD and DN-DISC1 may have affected intracellular trafficking of GLUT1, leading to accumulation of the transporter in the cytoplasm and an ensuing decrease in glucose uptake. Increased expression of *Slc2a1* could be a compensatory response. Similarly, consistent with decreased glucose uptake, *Ldha* (lactate dehydrogenase A), *Ldhb* (lactate dehydrogenase B), and *Pgk1* (phosphoglycerate kinase) expression is a compensatory response to diminished glucose uptake by primary astrocytes. Future studies will address the putative molecular mechanisms whereby DISC1 regulates glucose uptake.

Several lines of evidence suggest that brain bioenergetics are altered in schizophrenia and bipolar disorder^63^. Multiple reports have indicated reduced energy metabolism in the dorsolateral prefrontal cortex (DLPFC) in schizophrenia^64^, alterations in glucose homeostasis at the onset of the illness^65,66^, and impaired glucose tolerance in first-episode psychosis patients^67,68^. However, negative findings have also been reported^69^. Taken together, these results suggest that abnormal glucose metabolism may be associated with the pathogenesis and psychopathology of schizophrenia in the early phases of the disease^70^. Studies of patients with bipolar disorder have also found decreased high-energy phosphates, suggesting a shift toward glycolysis for energy production^71^, impaired oxidative phosphorylation and decreased total energy production and/or substrate availability^72^. Although we are not aware of any reports on metabolic abnormalities in members of the Scottish translocation family, our findings warrant future studies of this possibility.

The data indicate that a loss of function of DISC1 and resulting decreased lactate production could contribute to the behavioral changes observed in DN-DISC1 mice. Our results are consistent with growing evidence indicating that astrocytes play a critical role in energy supply traffic by up-taking glucose and producing lactate via glycolysis or glycogenolysis^40^. Notably, the astrocyte-neuron lactate shuttle hypothesis is not inconsistent with the fact that glucose also represents a major energy substrate in the brain and both metabolites are critical for proper brain functioning^73^. Our research is in line with a number of studies that demonstrate that brain lactate may influence learning and memory by maintaining an appropriate energy supply level during cognitive processing^74^. For example, direct intrahippocampal injections of lactate rescue working memory^74^ and passive avoidance training^35^. Pharmacological or genetic suppression of lactate transport in the brain inhibits long-term memory and decreases the rescue ability of lactate^35,74,75^. Our data are also in agreement with recent studies demonstrating that peripheral administration of lactate had antidepressant-like effects similar to those induced by desipramine^33^. We found that systemic acute injections of lactate increased time spent by DN-DISC1 mice in open arms of the EPM and decreased immobility time in the FST.

Our study does not address the regional effects of lactate metabolism on mouse behaviors. Although we assessed hippocampus-dependent behaviors, we do not suggest that this brain region exclusively mediates the behavioral abnormalities associated with DISC1 manipulations and/or pharmacological effects of lactate. Future studies will address these possibilities using regional genetic manipulation and/or lactate injections. We did not evaluate the potential neuronal changes in mice with reduced lactate production. Given that acute lactate treatment ameliorated the behavioral changes in DN-DISC1 mice, it is tempting to speculate that putative neuronal abnormalities are likely.

In conclusion, the present study demonstrates, for the first time, that DISC1 may be involved in the regulation of energy metabolism in astrocytes to support neuronal activity and associated behaviors. Altered expression of DISC1 in astrocytes leads to decreased glucose uptake and reduction of lactate production that could be responsible for affective and cognitive disorders consistent with aspects of major mental illnesses.

## Electronic supplementary material

supplemental tables

Supplemental Figure 1

Supplemental Figure 2

Supplemental Figure 3

Supplemental Figure 4

Supplemental Figure 5

Supplemental Figure 6

Supplemental Figure 7

Supplemental Figure 8

Supplemental Figure 9

Supplemental Figure 10

Supplemental Figure 11

Supplemental Figure 12

Supplemental Figure 13

Supplemental Figure 14

Supplemental methods and materials
