# Supplementary material for: DISC1 regulates lactate metabolism in astrocytes: implications for psychiatric disorders
Source: Transl Psychiatry. 2018 Apr 12;8:76. doi: 10.1038/s41398-018-0123-9 (PMC5895599; doi:10.1038/s41398-018-0123-9)
Supplement: Supplementary file 1 — supplemental tables [file 41398_2018_123_MOESM1_ESM.docx]

**Supplemental Table 1.** Primers used for qRT-PCR assyas

| Gene | Primers sequence | |
| --- | --- | --- |
|  | Forward | Reverse |
| *Slc2a1* | CTACTCAACGAGCATCTTCGAG | CCAATGAGGTGCAGGGTC |
| *Slc2a4* (set #1) | TTTTAAAACAAGATGCCGTCGG | ATAGCCAAACTGAAGGGAGC |
| *Slc2a4* (set #2) | GTGACTGGAACACTGGTCCTA | CCAGCCACGTTGCATTGTAG |
| *Slc16a3* | CTCTCAACTTCCAGCCTTCC | AACCATAGTGATCCTGCAGC |
| *Ldha* | TGGAAGACAAACTCAAGGGC | TGGAGTTCGCAGTTACACAG |
| *Ldhb* | AGCATTCTGGGAAAGTCTCTG | ACAATTTTCGGAGTCTGGAGG |
| *Pgk1* | AACCTCCGCTTTCATGTAGAG | GACATCTCCTAGTTTGGACAGTG |
| *Actin* | TGGAGAAGAGCTATGAGCTGCCTG | GTGCCACCAGACAGCACTGTGTTG |

Expression levels of *Slc2a4* were assessed using two different primers sets (1) and (2).

**Supplemental Table 2.** Expression of the genes involved in energy metabolism

| Gene | DISC1-KD | DN-DISC1 |
| --- | --- | --- |
| *Slc2a1* | 2.288 ± 0.0456 ↑ | 1.806 ± 0.0691 ↑ |
| *Slc2a4* (primers set #1) | 0.985 ± 0.041 | 0.412 ± 0.0154 ↓ |
| *Slc2a4* (primers set #2) | 0.840 ± 0.0691 | 0.499 ± 0.0586 ↓ |
| *Slc16a3* | 0.813 ± 0.0626 | 0.943 ± 0.0681 |
| *Ldha* | 0.065 ± 0.025 ↓ | 0.399 ± 0.0183 ↓ |
| *Ldhb* | 0.468 ± 0.0140 ↓ | 0.593 ± 0.134 ↓ |
| *Pgk1* | 0.345 ± 0.0153 ↓ | 0.387 ± 0.0231 ↓ |

Data are presented as mean ± SEM of normalized to *Actin* expression. Upwards arrows indicate a significant decrease; downwards arrows indicate a significant decrease.

Expression of *Slc2A1* (Glucose transporter 1) was significantly increased in DISC1-KD or DN-DISC1 primary astrocytes; two-tailed t-test with Bonferroni correction; p<0.05; n=5 individual samples per group.

Expression of *Slc2A4* (Glucose transporter 4) as assessed by two different primers sets, *Pygl* (Glycogen phosphorylase), *Ldha* (Lactate dehydrogenase A), *Ldhb* (Lactate dehydrogenase B), and *Pgk1* (Phosphoglycerate kinase) was decreased in DISC1-KD or DN-DISC1 primary astrocytes; two-tailed t-test with Bonferroni correction; p<0.05; n=5 individual samples per group.

Unaltered expression of *Slc16a3* (Monocarboxylate transporter 4) was found in DISC1-KD or DN-DISC1 primary astrocytes, n=5 individual samples per group.
