# Supplementary figures and images for: DISC1 regulates lactate metabolism in astrocytes: implications for psychiatric disorders
Source: Transl Psychiatry. 2018 Apr 12;8:76. doi: 10.1038/s41398-018-0123-9 (PMC5895599; doi:10.1038/s41398-018-0123-9)

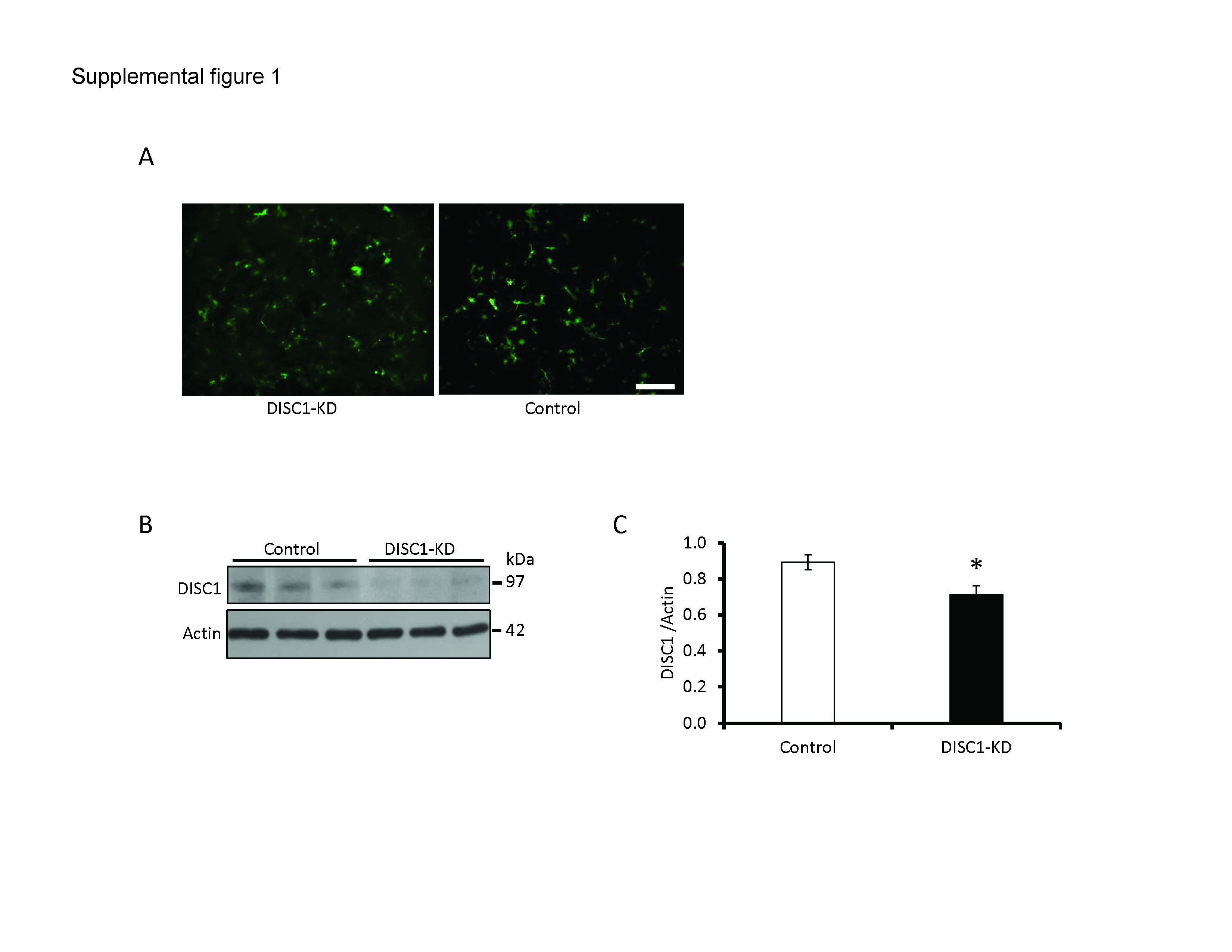

Supplement: Supplementary file 2 — Supplemental Figure 1 [file 41398_2018_123_MOESM2_ESM.tif]

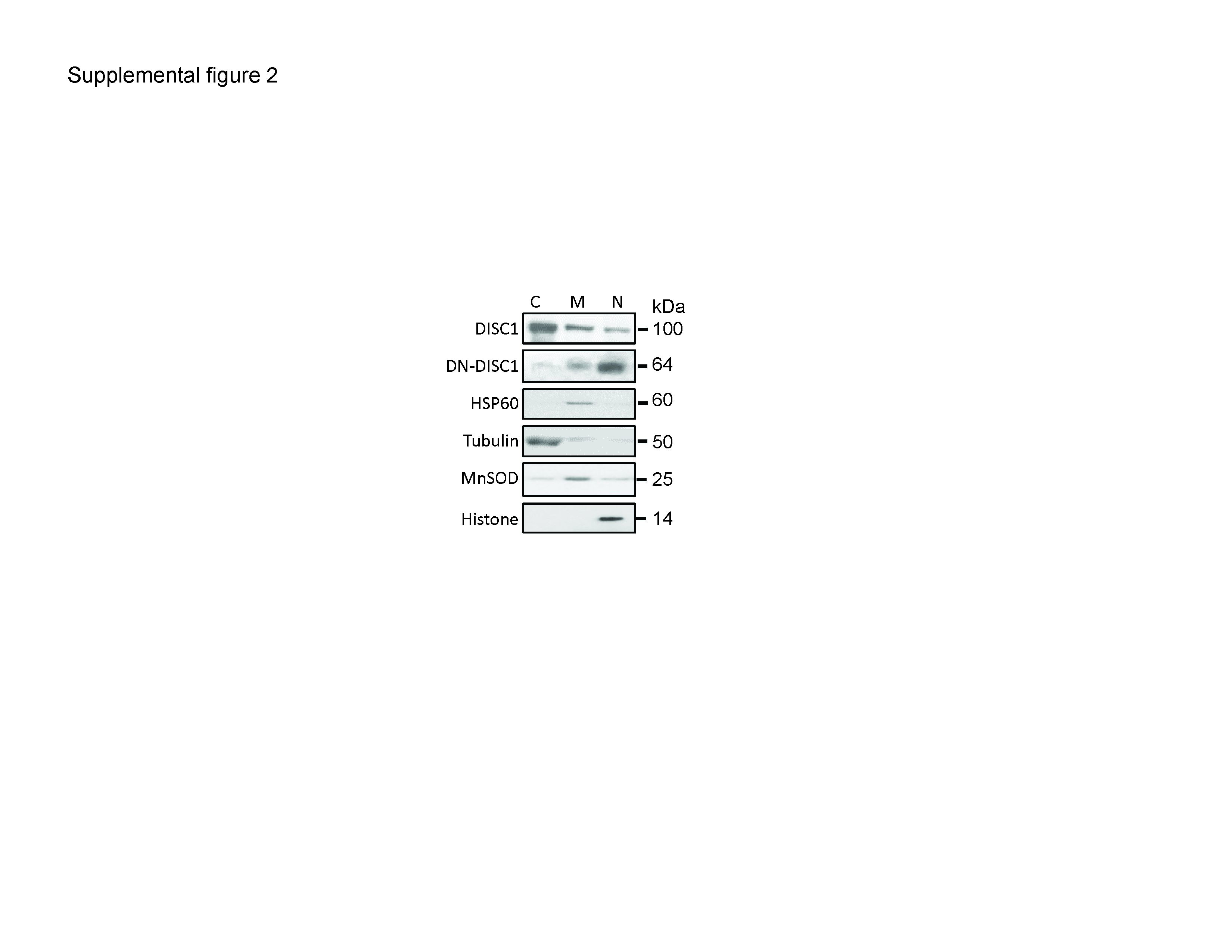

Supplement: Supplementary file 3 — Supplemental Figure 2 [file 41398_2018_123_MOESM3_ESM.tif]

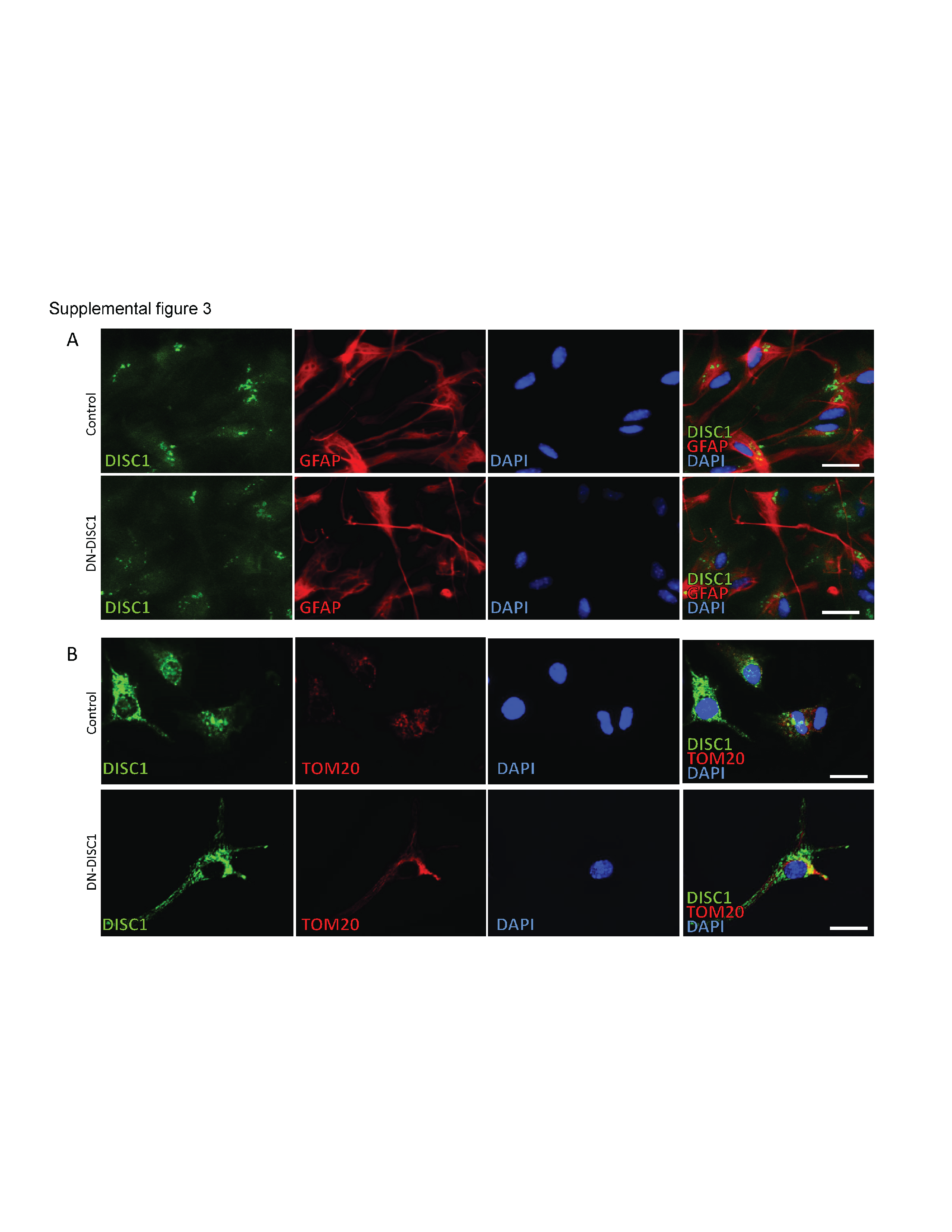

Supplement: Supplementary file 4 — Supplemental Figure 3 [file 41398_2018_123_MOESM4_ESM.tif]

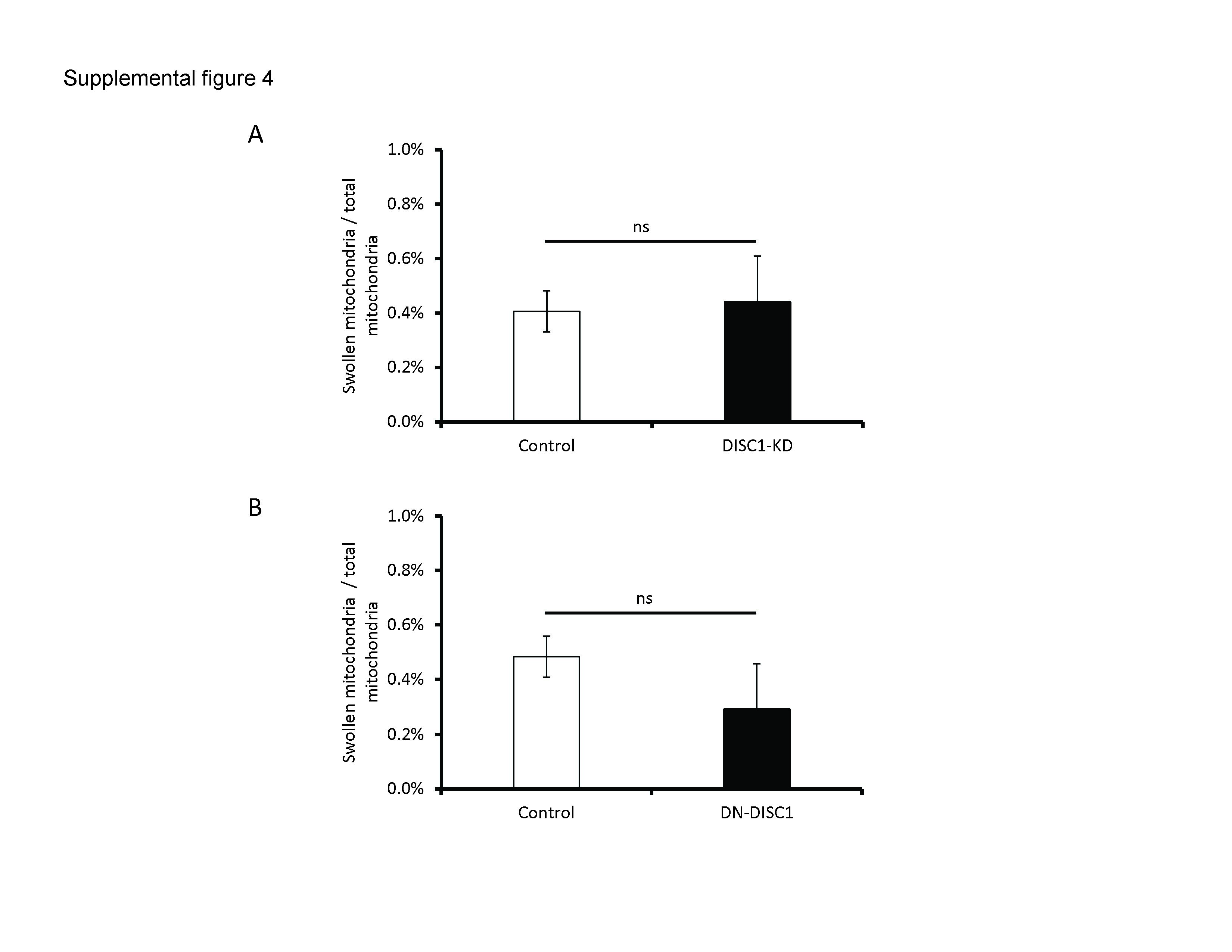

Supplement: Supplementary file 5 — Supplemental Figure 4 [file 41398_2018_123_MOESM5_ESM.tif]

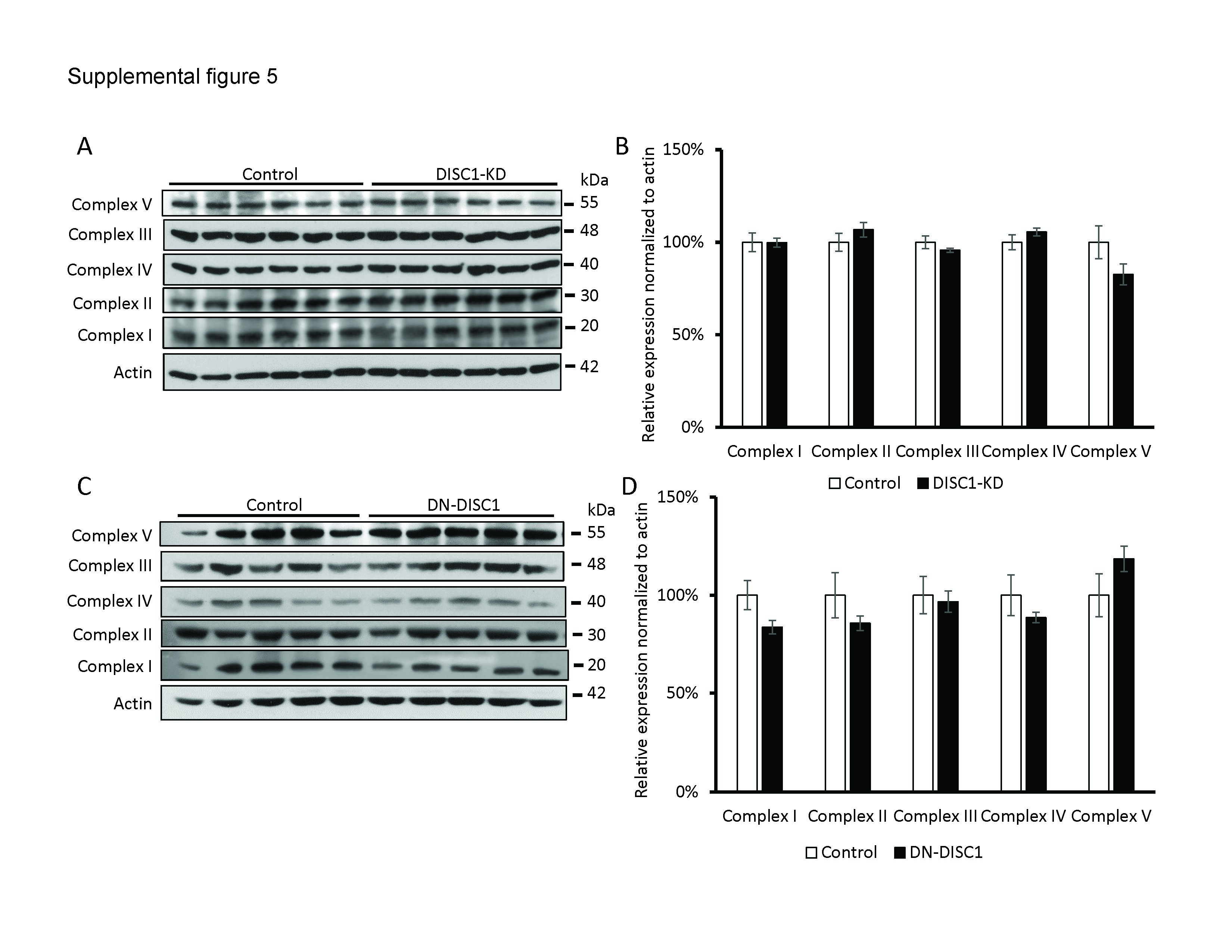

Supplement: Supplementary file 6 — Supplemental Figure 5 [file 41398_2018_123_MOESM6_ESM.tif]

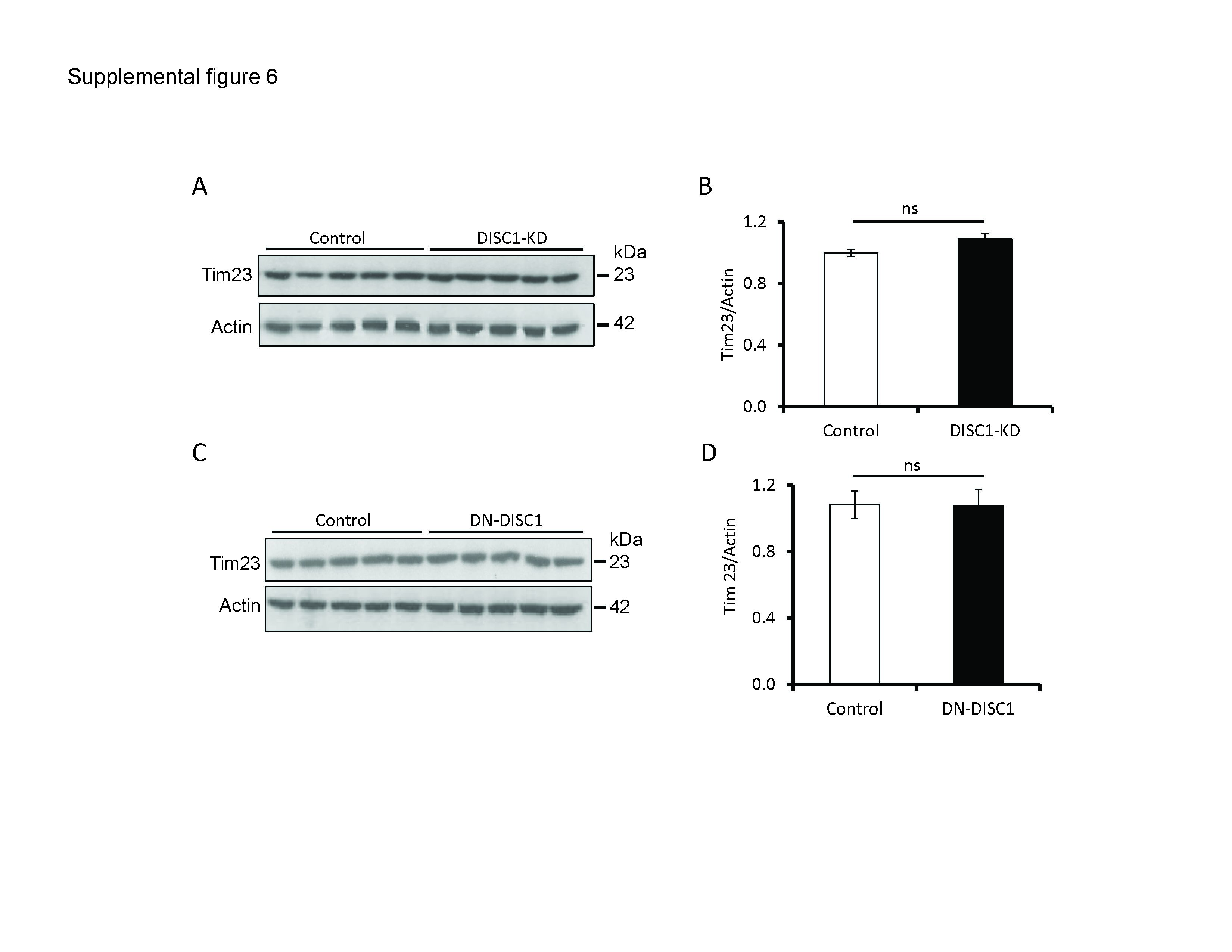

Supplement: Supplementary file 7 — Supplemental Figure 6 [file 41398_2018_123_MOESM7_ESM.tif]

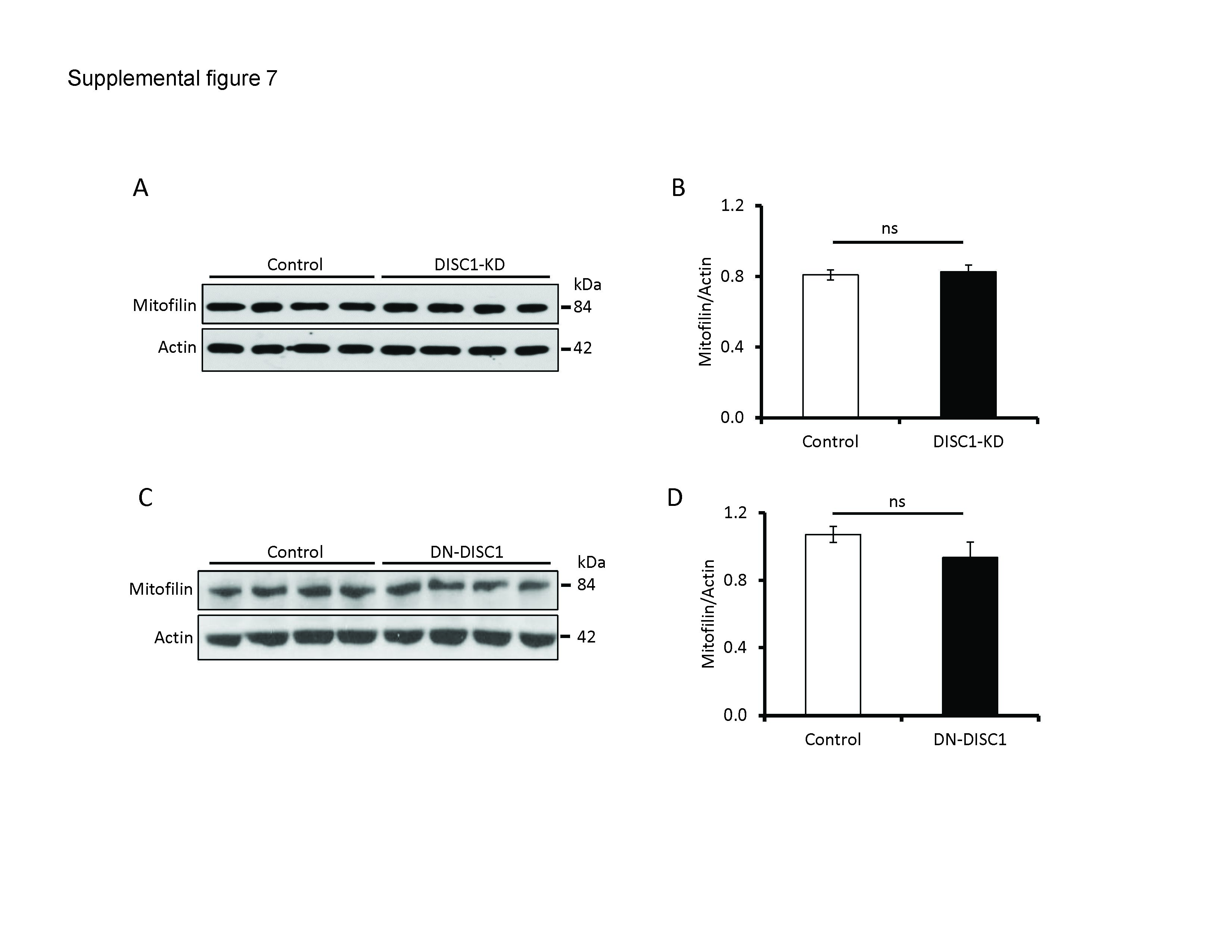

Supplement: Supplementary file 8 — Supplemental Figure 7 [file 41398_2018_123_MOESM8_ESM.tif]

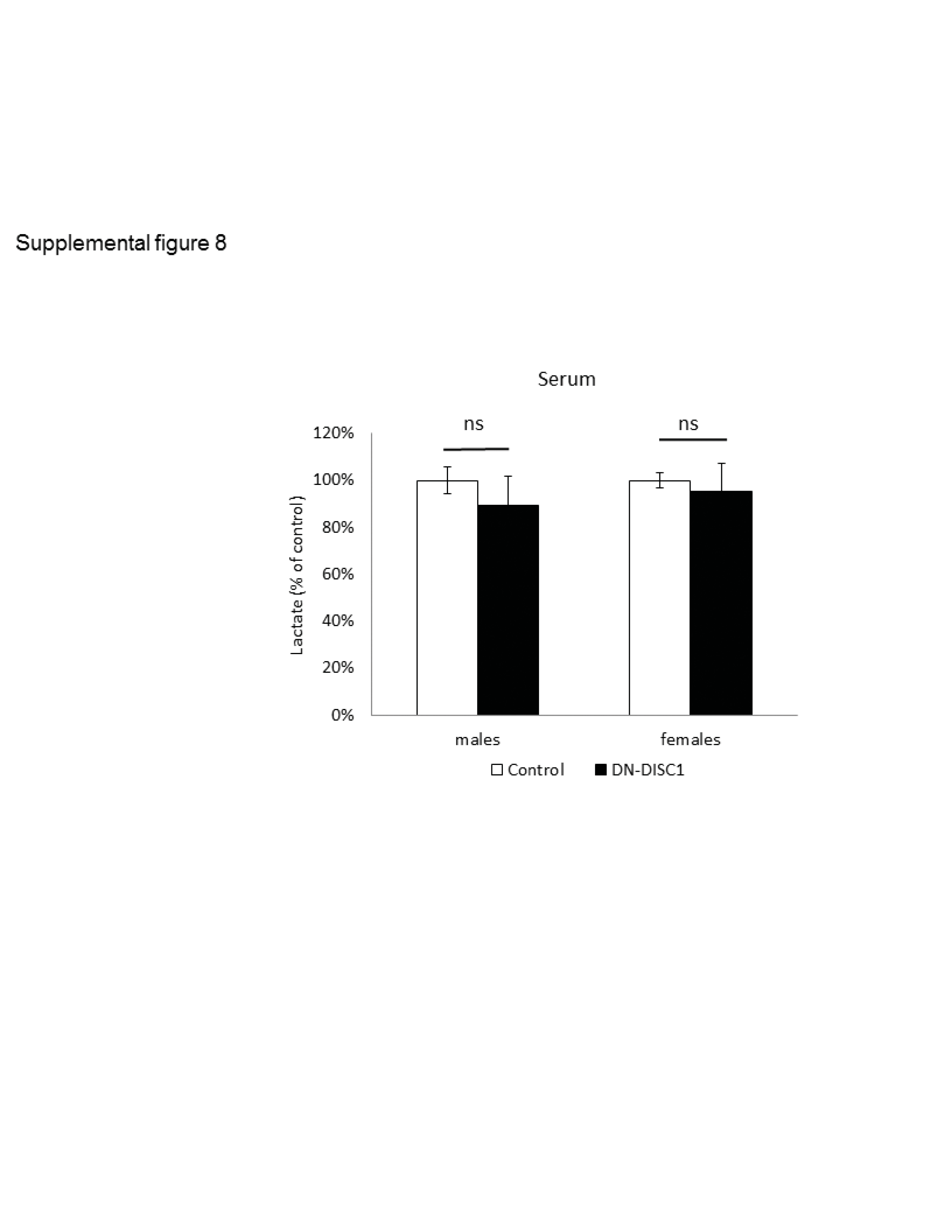

Supplement: Supplementary file 9 — Supplemental Figure 8 [file 41398_2018_123_MOESM9_ESM.tif]

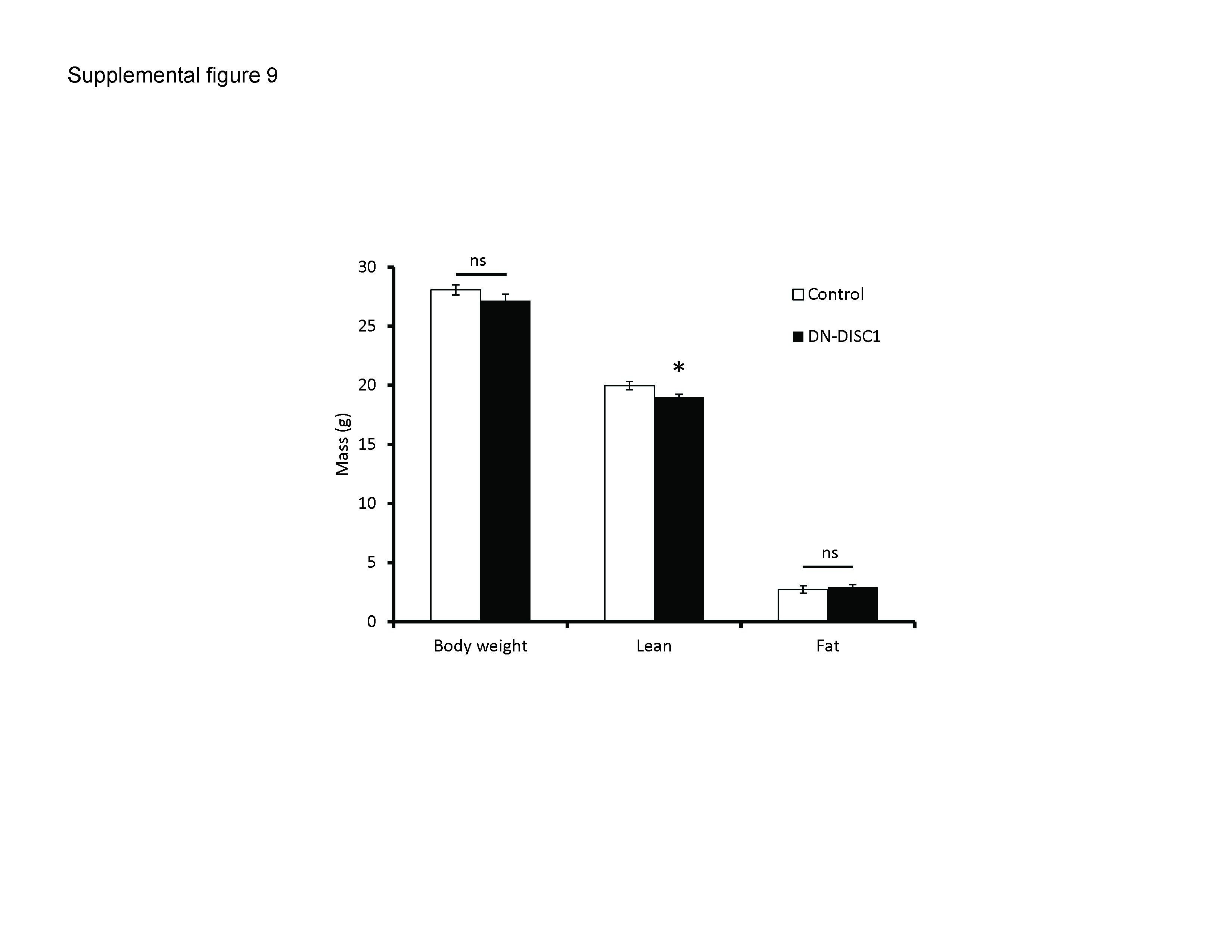

Supplement: Supplementary file 10 — Supplemental Figure 9 [file 41398_2018_123_MOESM10_ESM.tif]

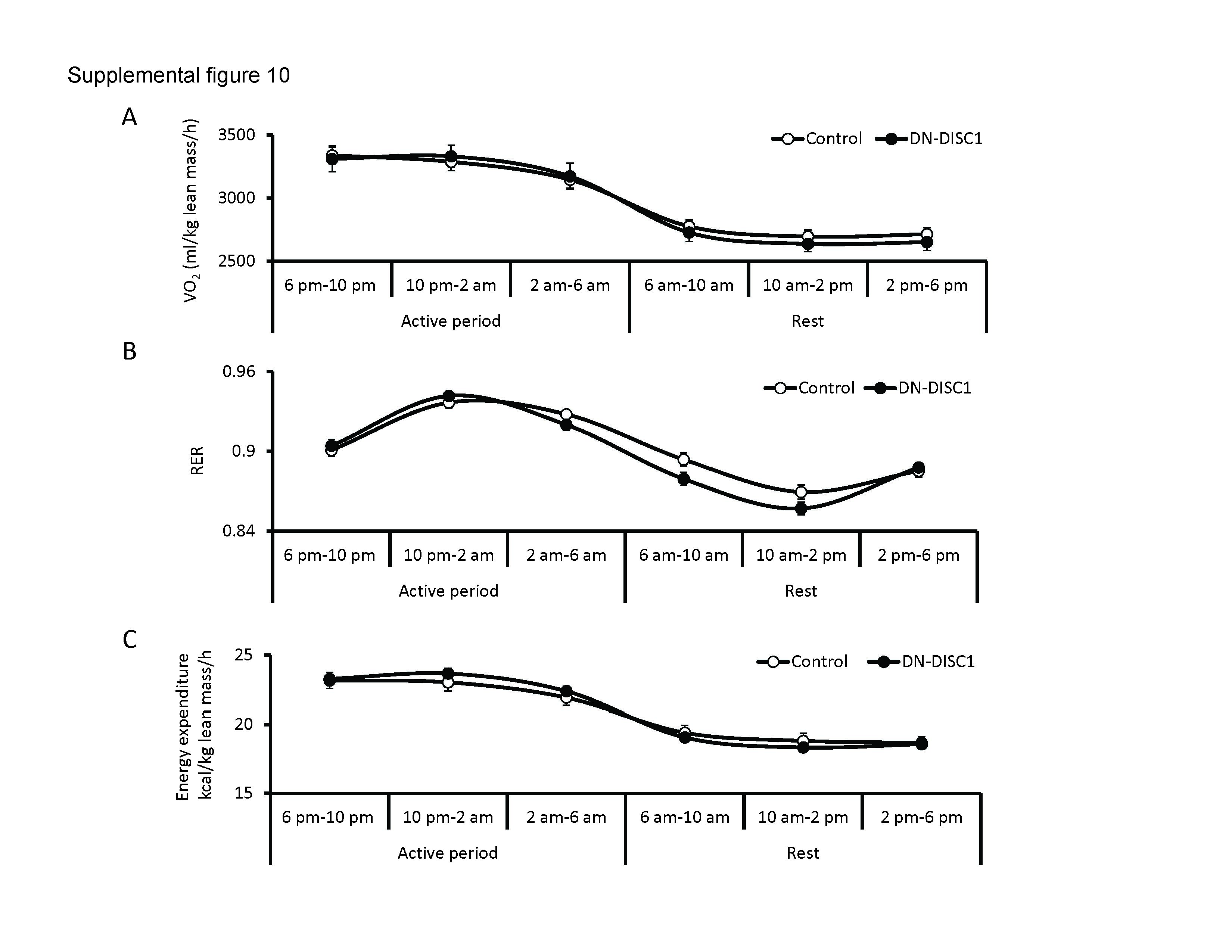

Supplement: Supplementary file 11 — Supplemental Figure 10 [file 41398_2018_123_MOESM11_ESM.tif]

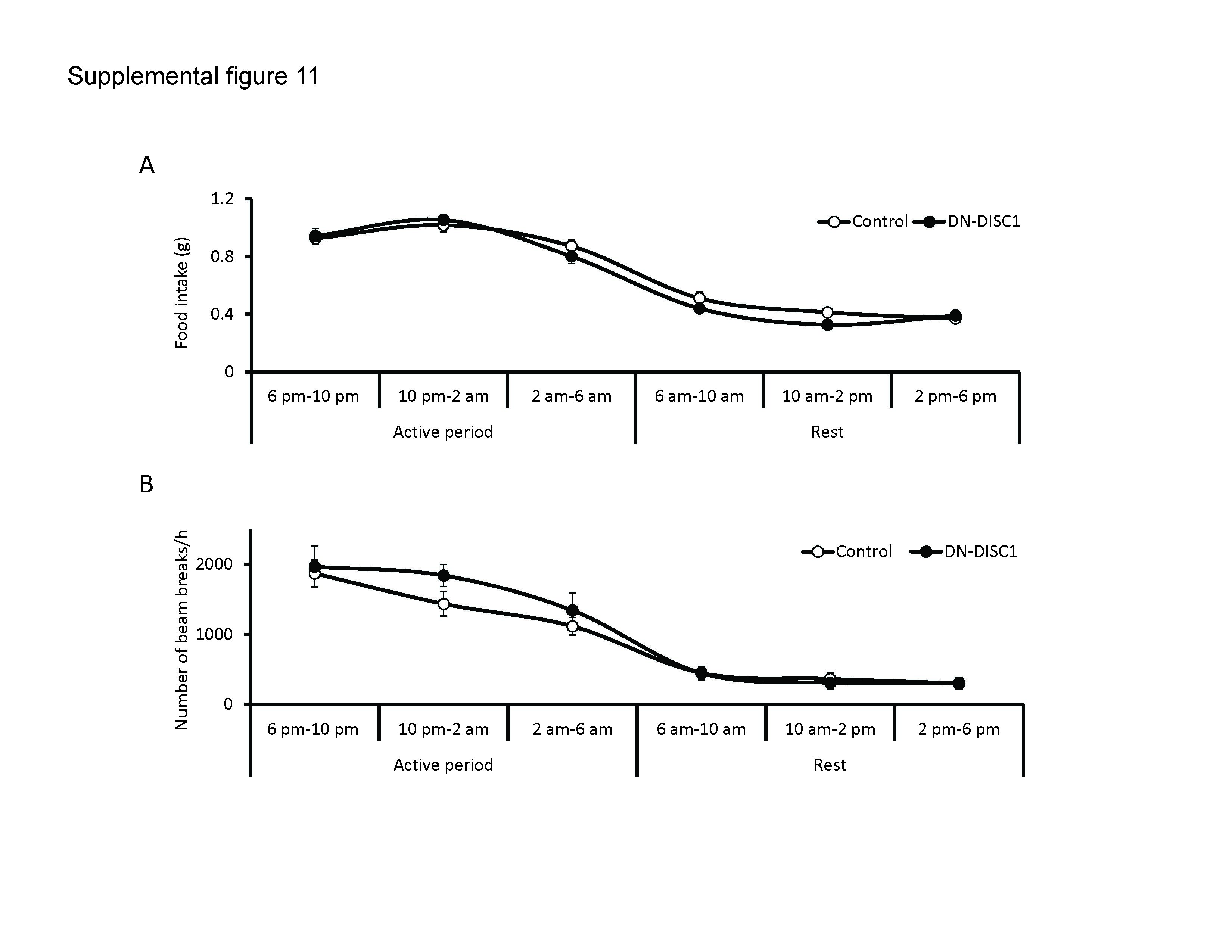

Supplement: Supplementary file 12 — Supplemental Figure 11 [file 41398_2018_123_MOESM12_ESM.tif]

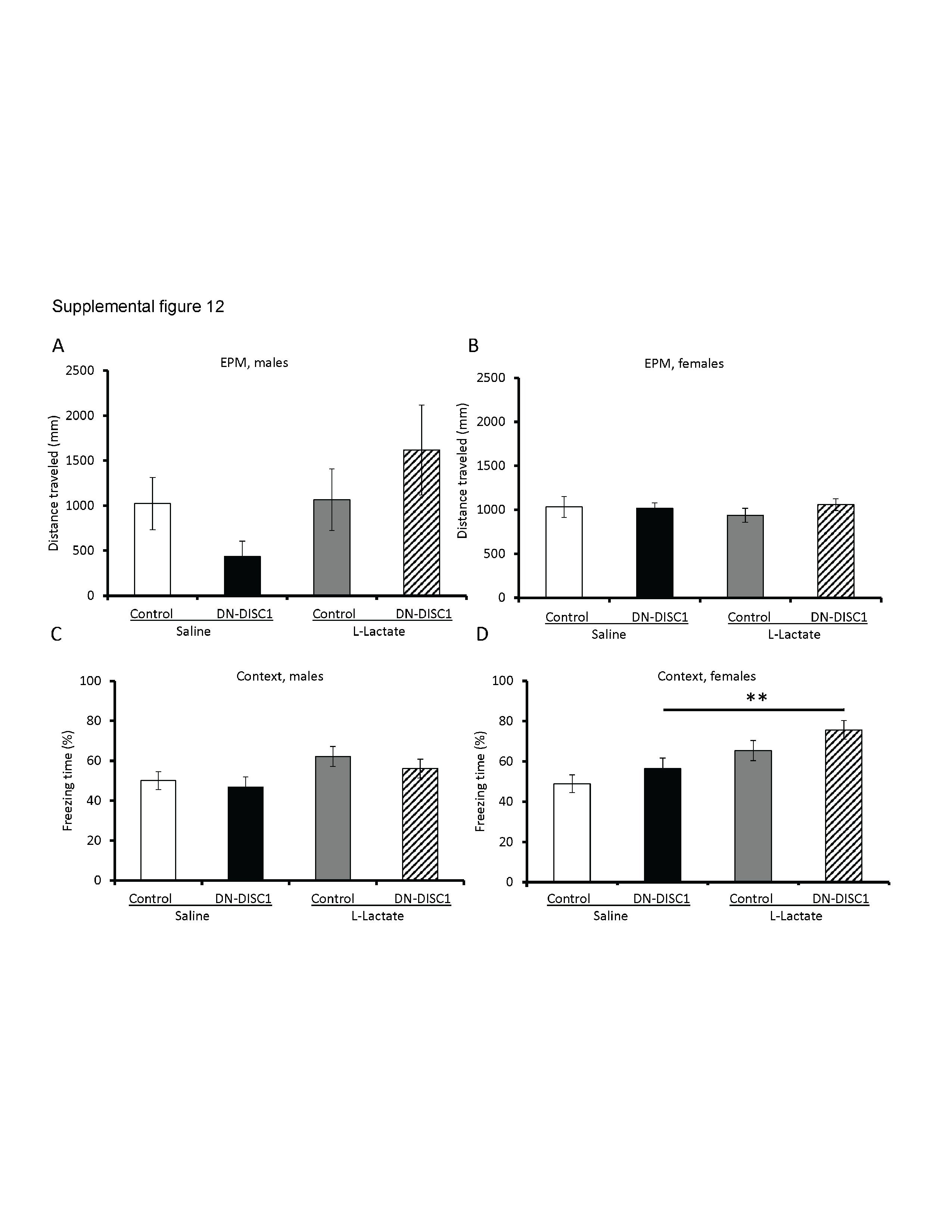

Supplement: Supplementary file 13 — Supplemental Figure 12 [file 41398_2018_123_MOESM13_ESM.tif]

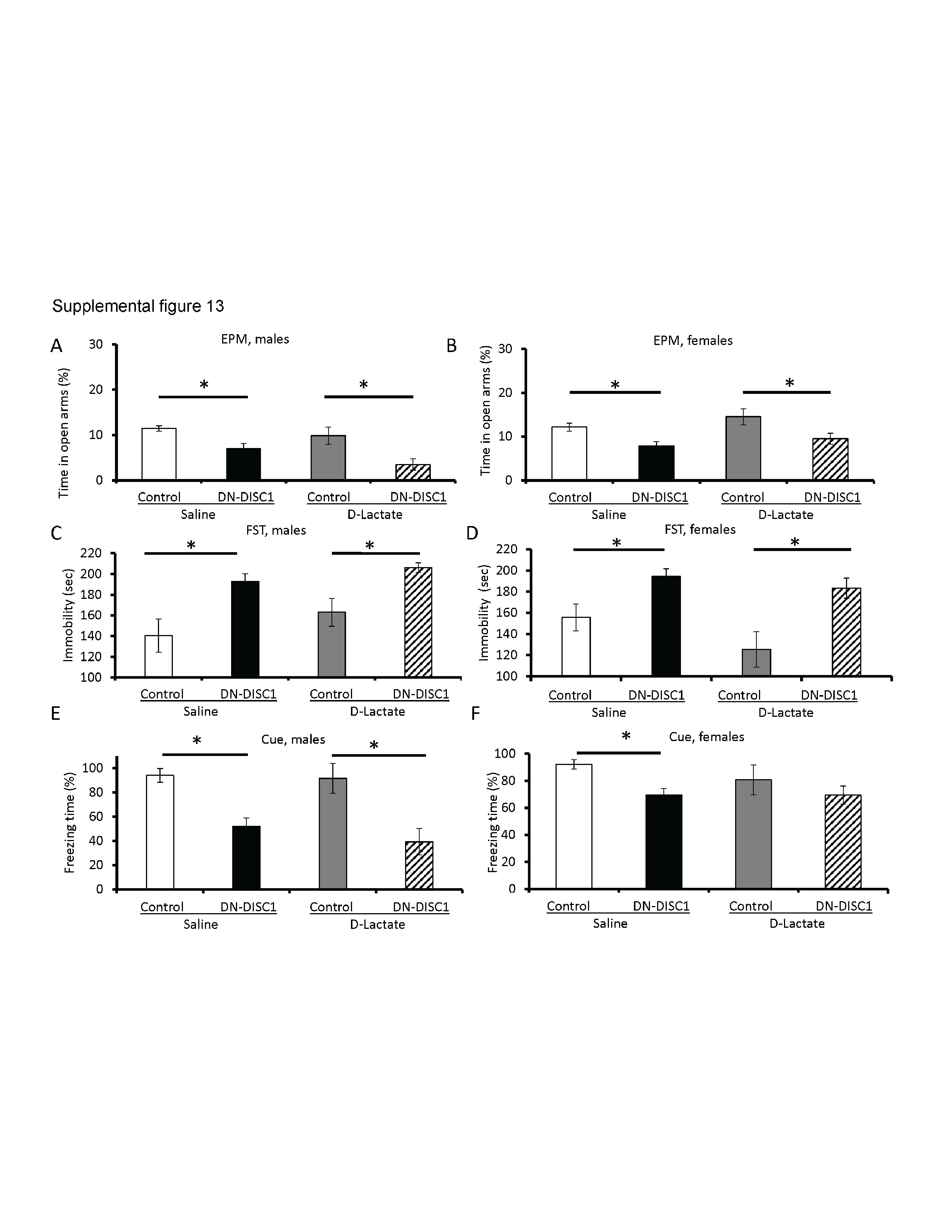

Supplement: Supplementary file 14 — Supplemental Figure 13 [file 41398_2018_123_MOESM14_ESM.tif]

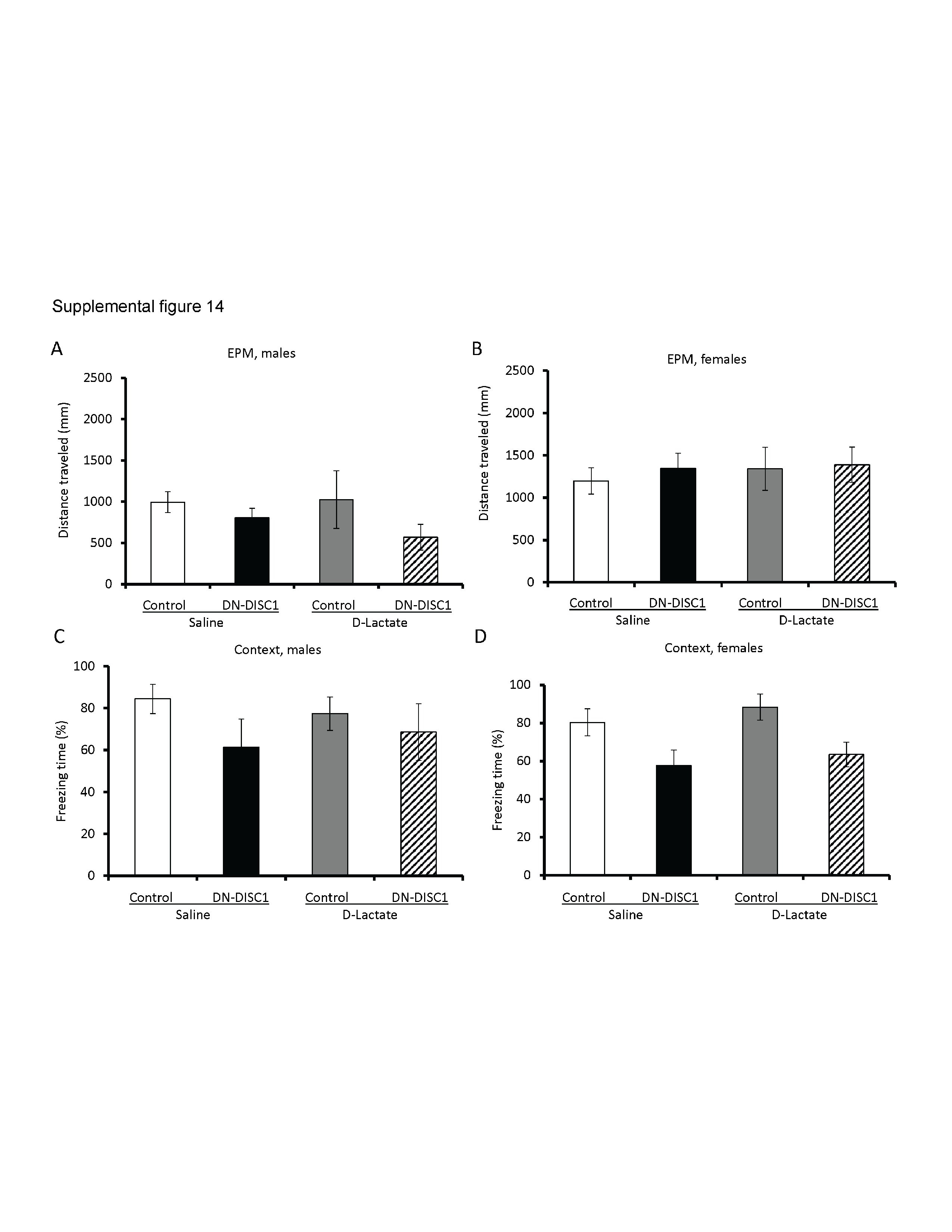

Supplement: Supplementary file 15 — Supplemental Figure 14 [file 41398_2018_123_MOESM15_ESM.tif]
