# Supplementary material for: DISC1 regulates lactate metabolism in astrocytes: implications for psychiatric disorders
Source: Transl Psychiatry. 2018 Apr 12;8:76. doi: 10.1038/s41398-018-0123-9 (PMC5895599; doi:10.1038/s41398-018-0123-9)
Supplement: Supplementary file 16 — Supplemental methods and materials [file 41398_2018_123_MOESM16_ESM.docx]

**Supplemental Methods and Materials**

*Primary Astrocytes*

Primary hippocampal astrocytes were prepared from one-day-old mouse pups as previously described ^[1](#_ENREF_1)^. Briefly**,** mice were sacrificed by quick decapitation after spraying the head and neck of each mouse pup with 70% ethanol. Hippocampi were dissected out, placed in ice-cold Dulbecco's phosphate-buffered saline (Thermo Fisher Scientific, 1190144) and dissociated with 0.25% trypsin-EDTA (Thermo Fisher Scientific, 25200056) at 37 °C for 15 minutes with shaking every 5 min. Cells were collected by centrifugation for 5 min at 300 x g and dissociated by vigorous pipetting using a 10-ml plastic pipette. Cells were plated in 6-well plates and incubated using astrocyte culture medium containing Dulbecco’s modified Eagle’s medium, high glucose (DMEM) (Thermo Fisher Scientific, 11995073) supplemented with 10% heat-inactivated fetal bovine serum (FBS) (Sigma-Aldrich, F4135), and 1% penicillin-streptomycin (Sigma-Aldrich, P0781). Cells were grown in a humid atmosphere of 5% CO_2_ at 37 °C. The medium was changed the next day and every other day thereafter. To remove loosely attached microglial cells and oligodendrocyte precursor cells before medium change, plates were shaken vigorously by hand for 2-3 min. After 8-12 days in culture, the glial cultures were passaged using trypsin and allowed to form a confluent monolayer. Primary cultures consisted of approximately 90-95% astrocytes, as evaluated by immunostaining with anti-GFAP antibody (data not shown). Based on cell morphology, most other cells in primary cultures were microglia.

*DISC1 Knockdown in Primary Astrocytes*

C57BL/6J wild-type primary astrocytes were transduced with lentiviruses carrying either *DISC1* shRNA or scrambled shRNA as described in our previous studies ^[2](#_ENREF_2)^. Specifically, we used DISC1 shRNA (5’-GGCAAACACTGTGAAGTGC-3’) and scrambled control shRNA (5’-GGAGCAGACGCTGAATTAC-3’). The lentivirus particles were produced by transient transfection of 293FT cells (Thermo Fisher Scientific, R70007, mycoplasma negative) with the packaging plasmids psPAX.2 (Addgene, 12260,) and pMD2.G (Addgene, 12259) at a ratio of 6:2:1 using Lipofectamine 2000 (Thermo Fisher Scientific, 11668-019). High-titer lentivirus (>10^9^ TU/ml) was then used for transductions. Transduction efficacy was tested after 4 days by counting GFP-positive cells. All assays in this study were performed 3-4 days after DISC1-KD treatment and resulted in 40% transduced cells.

*Expression of DN-DISC1 in Primary Astrocytes*

To prepare primary astrocytes that express DN-DISC1, we used one-day-old mouse pups generated by crossing congenic C57BL/6J single hemizygous transgenic GFAP-tTA mice with congenic C57BL/6J single homozygous transgenic TRE-mutant DISC1 mice as described previously ^[3](#_ENREF_3)^. This mating protocol produces litters that have ~50% single transgenic mice that do not express DN-DISC1 (control mice) and ~50% double transgenic mice that express DN-DISC1 (DN-DISC1 mice). Tail tissue samples were used to determine the genotype of mouse pups used for primary astrocyte cultures as previously described ^[3](#_ENREF_3)^.

*Immunocytochemistry*

Primary astrocytes were passed once and allowed to grow until 70% confluence. Astrocytes were then fixed in 4% paraformaldehyde (PFA) and stained with mouse anti-GFAP antibody (1:500, GA5 mouse mAb #3670, Cell Signaling), mouse anti-TOM20 (1:500, sc-17764, Santa Cruz Biotechnology) or rabbit anti-mouse DISC1 antibody (1:500, Zymed) followed by overnight incubation with Alexa Fluor® 488 Goat anti-Rabbit (1:1000, A-11008, Life technologies) or Alexa Fluor® 568 Goat anti-mouse secondary antibodies (1:1000, A-11004, Life technologies). Images were obtained using a confocal microscope (LSM 510 Meta, Carl Zeiss).

*Measurement of Mitochondrial Membrane Potential (ΔΨ) and Mitochondrial Morphology*

To determine the effects of DISC1-KD or DN-DISC1 on the morphology of mitochondria or the membrane potential, primary astrocytes were incubated with 100 nM of the membrane-potential-independent MitoTracker Green (MTG) (Thermo Fisher Scientific, M7514,) and 8 nM of the membrane-potential-dependent tetramethylrhodamine, ethyl ester (TMRE) (Thermo Fisher Scientific, T669) for 30 minutes. Afterwards, cells were gently washed three times with PBS and incubated with a live cell imaging solution (Thermo Fisher Scientific, A14291DJ). Live cells were imaged using a confocal microscope (LSM 510 Meta, Carl Zeiss) with a 100X oil-immersion objective. MTG was excited with a 488-nm argon laser, and emission was recorded with a 500- to 550-nm bandpass filter. TMRE was excited with a 543-nm helium-neon laser, and emission was recorded with a 650- to 710-nm bandpass filter. Data were analyzed and are presented as the TMRE/NTG ratio. The ratio of the area occupied by swollen, spherical mitochondria with reduced ΔΨ [^4^](#_ENREF_4) to the area occupied by all mitochondria in a given astrocyte was calculated. At least 20 cells were evaluated for each group.

*Measurements of Oxygen Consumption Rate*

The mitochondrial oxygen consumption rate (OCR) was assessed in primary astrocytes using an XF24 Extracellular Flux Analyzer (Seahorse Bioscience) as described previously ^[5](#_ENREF_5)^. Briefly, approximately 100,000 primary astrocytes were plated at a density of 50 × 10^3^ per well in XF24 cell-culture microplates pre-coated with poly-D-lysine (Sigma-Aldrich, P6407). The cultures were incubated for 24 hours in growth medium. The next day, the medium was replaced with Seahorse XF medium supplemented with 10 mM glucose (Sigma-Aldrich, G8769), 1 mM L-glutamine (Thermo Fisher Scientific, 25030081) and 1 mM sodium pyruvate (Thermo Fisher Scientific, 11360070). OCR was analyzed in an XF24 analyzer after 45 min of incubation at 37 °C in a CO_2_-free incubator. The first OCR measurement was recorded following 11-min equilibration, a 1-min mix period, and a 1-min wait period. Oligomycin, carbonyl cyanide m-chlorophenylhydrazone, (CCCP) and rotenone were sequentially added to each well to assess basal respiration, coupling of the respiratory chain, and mitochondrial respiratory capacity, respectively.

*Measurement of Glycolysis as Extracellular Acidification Rate*

Primary astrocytes were plated at a density of 50 × 10^3^ per well in XF24 cell-culture microplates for 48 hours. On the day of assay, the medium was replaced with glucose-free XF24 Seahorse medium. Glycolytic flux (basal glycolysis, glycolytic capacity, and glycolytic reserve) was assessed as the extracellular acidification rate (ECAR) by sequentially adding glucose, oligomycin, and 2-deoxyglucose to the XF24 flux analyzer ^[6](#_ENREF_6)^. ECAR was measured at 37 °C with a 1-min mix, 1-min wait, and 2-min measurement protocol. Seahorse analysis was started after 45 min of incubation in a CO_2_-free incubator. The first ECAR measurement was recorded after 11-min equilibration, a 1-min mix period, and a 1-min wait period.

*Glucose Uptake Assay*

Glucose uptake was measured in culture medium using a glucose uptake cell-based assay kit (Cayman CHEMICAL, 600470). Primary astrocytes were seeded the day before at a density of 10,000 cells per well in 96-well plates. The fluorescently labeled glucose analog 2-N-(7-nitro-benz-2-oxa-1,3-diazol-4-yl) amino)-2 deoxyglucose (2-NBDG) was used to measure glucose uptake. Primary astrocytes were incubated with 2-NBDG for 30 min and then washed three times with phosphate-buffered saline (PBS). Cell lysate was used to detect the fluorescence signal at the excitation and emission wavelengths of 485 nm and 535 nm, respectively, using a GloMax Discover System luminometer (Promega) in a 96-well microplate.

*RNA extraction and RT-PCR*

DN-DISC1 and control primary astrocytes were detached by trypsin treatment and rinsed in PBS. Cell pellets were then re-suspended in Tri-reagent and total RNA extracted as per the manufacturer’s instructions (T9424, Sigma-Aldrich). RNA was quantified by Nanodrop (Thermo Fisher Scientific); 500 ng of RNA was subjected to DNase treatment (18068015, Thermo Fisher Scientific) as per the manufacturer’s instructions. First-strand cDNA was synthesized using Superscript III (18080051, Thermo Fisher Scientific) as described by the manufacturer. For quantitative PCR (qPCR) amplification, cDNA corresponding to 50 ng of cDNA was obtained using SsoFast EvaGreen Supermix (BIO-RAD, 172-5201), as described by the manufacturer. The primers’ sequences are presented in Supplemental Table 1. qPCRs were performed on an ABI7900HT real-time PCR system (Applied Biosystems). The results were analyzed with SDS 2.4 software (version 2.4; Applied Biosystems). We used *Actin* for normalization of mRNA expression levels of the target genes.

*Western Blotting*

For Western blot assays, 2-month-old control and DN-DISC1 mice were euthanized, and their hippocampi were isolated on ice, immediately frozen and stored at -80 °C until protein extraction. Primary astrocytes were collected by directly adding cell lysis buffer (Cell Signaling Technology, 9803) to cell plates, followed by incubation on dry ice for 5 min. Cells were scraped from the plates using a cell scraper and stored at -80 °C. Isolated hippocampi or collected primary astrocytes were sonicated for 30 s in cell lysis buffer (Cell Signaling Technology, 9803) containing 1 mM EDTA, 1 mM EGTA, 1% Triton, 2.5 mM sodium pyrophosphate, 1 mM sodium orthovanadate, 1 mM PMSF and 1 μg/mL leupeptin. Cell lysates were spun down at 10,000 × g for 10 min at 4 °C. The resulting supernatant was subjected to SDS/PAGE, and the separated proteins were transferred onto a nitrocellulose membrane. The membrane was washed in Tris-buffered saline solution containing 0.05% Tween 20 (TBST) and was blocked for 1 hour at RT in TBST containing 5% non-fat dry milk. The membrane was incubated overnight at 4 °C with a primary antibody. Membranes were probed with custom-made rabbit anti-DISC1 antibody (1:500) [^7^](#_ENREF_7) to assess the expression of endogenous DISC1 protein and with rabbit anti-myc antibody to assess the expression of DN-DISC1 tagged with c-*myc* (1:500, 11667149001, Roche), Tim23 antibody (1:1000, 611222; BD Biosciences), mitofilin antibody (1:1000, Novus Biologicals, NB100-1919SS), OXPHOS antibody cocktail for NADH dehydrogenase [ubiquinone] 1 beta subcomplex subunit 8 (NDUFB8) (α-subunit of complex I), succinate dehydrogenase (complex II), Core2 (complex III subunit), cytochrome c oxidase (complex IV), α-subunit of complex V (1:500, ab110413, MitoSciences), anti-glucose transporter GLUT-4 (1:1000, PA5-23052, Thermo Fisher Scientific), anti-glucose transporter GLUT-1 antibody (1:1000, 07-1401, Millipore-Sigma), and mouse anti-β-actin (1:2000, A5441, Sigma-Aldrich). After being washed with TBST three times, membranes were incubated with HRP-conjugated donkey anti-rabbit IgG (1:1000, NA934V, GE Healthcare) or donkey anti-mouse IgG (1:1000, NXA931, GE Healthcare) secondary antibodies for one hour at room temperature. The immunoblots were visualized in Blu-Ray autoradiography films (NDA8803, Next Day Science) and Super Signal West Pico Chemiluminescent Substrate (34080, Thermo Fisher Scientific). The optical density of protein bands on each digitized image was normalized to the optical density of the β-actin using the freely available ImageJ software program (version 1.49v).

*Lactate Measurement in Primary Astrocytes*

Lactate was measured in medium using an L-lactate assay kit (MAK064, Sigma-Aldrich). Cells were seeded at a density of 20,000 cells per well in 96-wells plates pre-coated with poly-D-lysine. The next day, the medium was replaced with FBS-free medium (Thermo Fisher Scientific, 11965092). After one hour of incubation, the medium was collected and centrifuged at 10,000 x g for 5 minutes. The secreted L-lactate concentration in the supernatant was determined by absorbance measurement at 570 nm. We normalized the values to the number of cells in the well.

*Indirect Calorimetry and Energy Balance Measurements*

To determine whether DN-DISC1 expression in astrocytes affects whole-body metabolism, male DN-DISC1 (n=10) and control (n=13) mice were monitored individually in an open-circuit indirect calorimeter with additional features to measure ad libitum food intake and physical activity (Comprehensive Lab Animal Monitoring System, CLAMS, Columbus Instruments, Columbus OH). Mice were monitored for four consecutive days, the first three of which involved confirming behavioral and metabolic adaptation. Rates of O_2_ consumption (VO_2_) and CO_2_ production (VCO_2_) were measured every 24 min per mouse and input into software (Oxymax V.4.93) to calculate the respiratory exchange ratios (RER=VCO2 / VO2) and rates of energy expenditure (EE) [^10^](#_ENREF_10). Oxymax calorimetry data outputs were on a per-kg body weight basis, then renormalized to estimate per-kg-lean mass, utilizing body composition data from the mice measured in an EchoMRI-100 immediately prior to CLAMS. Calorimetry data, as well as food intake and physical activity data, are presented in 4-hr bins of averaged data per mouse, averaged for each group.

*Lactate Assay in Brain Tissue and Blood*

We measured lactate levels in the hippocampus and blood serum of control and DN-DISC1 two-month-old male mice. Mice were sacrificed, and their hippocampi and blood samples were collected and processed for lactate measurements according the manufacturer’s method (Sigma-Aldrich, MAK065). We normalized the values to protein concentrations in the brain tissue or serum samples.

*Behavioral Tests*

Behavioral tests were performed on 2-month-old control and DN-DISC1 male and female mice. The elevated plus maze (EPM), the forced swim test (FST) and trace fear conditioning (TFC) were used to assess the behavioral effects of preferential L-lactate administration. The EPM and FST were described in detail in our previous publications[^11-14^](#_ENREF_11). TFC was a three-day test consisting of a habituation day, training day, and test day. Mice were habituated to a shock box (Coulbourn Instruments, Holliston, MA) for 10 minutes. The following day, mice were placed in the shock box, and a 20-s white noise tone was delivered. Twenty seconds following the termination of the tone, a scrambled 2-s 0.5-mA shock was delivered. This tone-shock pairing was repeated 3 times. On the third day, mice were placed in the shock box for three minutes to measure freezing in response to the context. Following the exposure to the shock box, mice were placed in a different box (a new context not associated with the shock), and the 20-s white noise tone was delivered, during which freezing behavior in response to the tone was measured.

*Lactate Treatments*

Mice received single intraperitoneal (ip) injections of vehicle or L-lactate (1 mg/kg) or D-lactate (1 mg/kg) one hour prior to testing.

**Supplemental Figures**

**Supplemental Figure 1. Validation of *DISC1* knockdown in primary astrocytes.**

Validation of transduction efficacy of primary hippocampal astrocytes with lentiviruses (LV) carrying shRNA for *DISC1*. Approximately 40% of primary astrocytes were successfully transduced with LV carrying DISC1 shRNA (KD-DISC1) or scrambled (control) shRNA.

A - Representative images of DISC-KD (left) and control (right) primary astrocytes transduced with lentiviruses. Scale bar - 100 μm.

B – Representative images of the Western blotting bands corresponding to endogenous mouse DISC1 (~97 kDa) and actin used as a loading control.

C - Quantitative analysis of expression of mouse DISC1 following transduction of primary astrocytes with scrambled (Control) or DISC1 shRNA (DISC1-KD); 3 independent samples were collected from separate primary cultures; * denotes p<0.05 vs. control cultures; two-tailed Student’s t-test, t=2.807; data are presented as means ± SEM.

**Supplemental Figure 2. Expression of endogenous DISC1 and DN-DISC1 in the cellular compartments.**

Protein extracts from sub-cellular fractions of primary astrocytes were probed with the antibodies against the specific markers. Representative images of the Western blotting bands for the cytoplasmic (C), mitochondrial (M) and nuclear (N) fractions are shown. Endogenous mouse DISC1 (DISC1) and DN-DISC1 were present in all cellular compartments of primary astrocytes.

**Supplemental Figure 3. Localization of endogenous DISC1 and DN-DISC1 to mitochondria in astrocytes.**

A - Expression of endogenous mouse DISC1 (green) in control (upper panel) and DN-DISC1 (lower panel) primary GFAP^+^ (red) astrocytes; DAPI (blue) – nuclear staining; scale bar - 50 μm.

B – Co-localization of endogenous mouse DISC1 (green) with the mitochondrial marker TOM20 (red) in control (upper panel) and DN-DISC1 (lower panel) primary astrocytes; DAPI (blue) – nuclear staining; scale bar - 50 μm.

**Supplemental Figure 4. Quantification of abnormal mitochondria in primary astrocytes.**

**DISC1-KD**: A – The ratio of round mitochondria (counted as number of pixels) with reduced membrane potential to unaffected mitochondria was measured in 4 independent cultures (at least 20 cells per culture); ns - not significant; two-tailed Student’s t-test, t=0.861.

**DN-DISC1**: B – The ratio of round mitochondria (counted as number of pixels) with reduced membrane potential to unaffected mitochondria was measured in 4 independent cultures (at least 20 cells per culture); ns - not significant; two-tailed Student’s t-test, t=0.334; data are presented as means ± SEM.

**Supplemental Figure 5. Unaltered expression of electron transport chain subunits.**

**DISC1-KD**: A - Representative Western blotting images of expression of the subunits I-V by control and DISC1-KD primary astrocytes; B – quantitative analysis of the expression of the ETC subunits normalized to actin in control and DISC1-KD astrocytes; n=4 independent cultures; each culture was measured in triplicate; two-tailed Student’s t-test, t=0.0304 (Complex I), t=-1.070 (Complex II), t=1.172 (Complex III), t=-1.202 (Complex IV), and t=1.649 (Complex V).

**DN-DISC1:** C - Representative Western blotting images of expression of the subunits I-V by control and DN-DISC1 primary astrocytes; D – quantitative analysis of the expression of the ETC subunits normalized to actin in control and DN-DISC1 astrocytes; n=4 independent cultures; each culture was measured in triplicate; two-tailed Student’s t-test; t=1.991 (Complex I), t=1.182 (Complex II), t=t=0.302 (Complex III), t=- 1.064 (Complex IV), and t=-1.458 (Complex V); actin served as a loading control; data are presented as means ± SEM.

**Supplemental Figure 6. Unaltered expression of the protein import translocase Tim23.**

**DISC1-KD**: A - Representative Western blotting images of expression of Tim23 by control and DISC1-KD primary astrocytes; B – quantitative analysis of normalized expression of Tim23 by control and DISC1-KD astrocytes; n=4 independent cultures; each culture was measured in triplicate; two-tailed Student’s t-test, t=0.1; ns – not significant.

**DN-DISC1:** C - Representative Western blotting images of expression of Tim23 by control and DN-DISC1 primary astrocytes; D – quantitative analysis of normalized expression of Tim23 by control and DN-DISC1 astrocytes; n=4 independent cultures; each culture was measured in triplicate; two-tailed Student’s t-test, t=0.49; ns – not significant; actin served as a loading control; data are presented as means ± SEM.

**Supplemental Figure 7. No changes in expression of mitofilin.**

**DISC1-KD**: A - Representative Western blotting images of expression of mitofilin by control and DISC1-KD primary astrocytes; B – quantitative analysis of expression of mitofilin by control and DISC1-KD astrocytes; n=4 independent cultures; each culture was measured in triplicate; two-tailed Student’s t-test, t=0.347; ns - not significant.

**DN-DISC1:** C - Representative Western blotting images of expression of mitofilin by control and DN-DISC1 primary astrocytes; D – quantitative analysis of expression of mitofilin by control and DN-DISC1 astrocytes; n=4 independent cultures; each culture was measured in triplicate; two-tailed Student’s t-test, t=0.25; ns - not significant; actin served as a loading control; data are presented as means ± SEM.

**Supplemental Figure 8. No changes in blood lactate level in DN-DISC1 mice.**

Lactate level (mM) was measured in serum of control (open bars) and DN-DISC1 (solid bars) male and female mice; two-tailed Student’s t-test; t=0.769 (for males) and t=0.369 (for females); data are presented as means ± SEM.

**Supplemental Figure 9. No changes in body weight of DN-DISC1 mice.**

There were no significant genotype-dependent differences in body weight or fat mass. Lean mass was slightly decreased in DN-DISC mice; n=10-13 mice in each group; * denotes p<0.05, ns - not significant; two-tailed Student’s t-test, t=-2.178 for lean mass; data are presented as means ± SEM.

**Supplemental Figure 10. No changes in whole-body metabolism in DN-DISC1 mice.**

There were no significant genotype-dependent differences in oxygen consumption (A), the respiratory exchange ratio (B) or energy expenditure (C) during the active or rest periods, as assessed by indirect calorimetry; n=10-13 mice in each group; data are presented as means ± SEM.

**Supplemental Figure 11. No changes in food intake or locomotor activity in DN-DISC1 mice.**

There were no significant genotype-dependent differences in food intake (A) or spontaneous locomotor activity (B) in the indirect calorimetry chambers during the active or rest periods; n=10-13 mice in each group; data are presented as means ± SEM.

**Supplemental Figure 12. No effects of L-lactate on locomotor activity in EPM or context-dependent freezing in TFC.**

A-B - There were no significant effects of L-lactate treatment on locomotor activity in the EPM of male (A) or female (B) mice; n=4-6 male and 6-11 female mice in each group.

C - There were no significant effects of L-lactate treatment on context-dependent freezing in male mice; n=4-6 male in each group.

D – L-lactate significantly increased context-dependent freezing in DN-DISC1 female mice; n=7-15 female mice in each group. Two-way ANOVA showed a significant effect of treatment, F(1, 43)=10.55, P<0.01. A post hoc Bonferroni test showed that compared with saline-treated DN-DISC1 mice, lactate-treated DN-DISC1 mice exhibited significantly more context-dependent freezing (p=0.006); ** denotes p<0.01; data are presented as means ± SEM.

**Supplemental Figure 13. No significant effects of D-lactate on behavioral changes.**

Control and DN-DISC1 male and female 2-month-old mice were administered a single injection of D-lactate at a dose of 1 mg/kg (IP) 60 minutes before behavioral testing.

**EPM**: D-lactate administration did not alter the baseline genotype-dependent differences in time spent in open arms of the EPM in male (A) or female (B) mice; n=4-6 male and 6-11 female mice in each group.

*For male mice*, two-way ANOVA showed a significant effect of genotype, F(1,13)=14.92, P<0.01. Post hoc Bonferroni tests showed that compared with control mice (both treatments), DN-DISC1 mice (both treatments) spent significantly less time in open arms of the EPM (both p values<0.05).

*For female mice*, two-way ANOVA showed a significant effect of genotype, F(1,13)=14.43, P<0.01. Post hoc Bonferroni tests showed that compared with control mice (both treatments), DN-DISC1 mice (both treatments) spent significantly less time in open arms of the EPM (both p values<0.05).

**FST**: D-lactate administration did not alter the baseline genotype-dependent differences in the immobility time in male (C) or female (D) mice; n=6 male and n=7-11 female mice in each group.

*For male mice*, two-way ANOVA showed a significant effect of genotype, F(1,13)=14.37, P<0.01. Post hoc Bonferroni tests showed that compared with control mice (both treatments), DN-DISC1 mice (both treatments) exhibited greater immobility in the FST (both p values<0.05).

*For female mice*, two-way ANOVA showed a significant effect of genotype, F(1,13)=16.31, P<0.01. Post hoc Bonferroni tests showed that compared with control mice (both treatments), DN-DISC1 mice (both treatments) exhibited greater immobility in the FST (both p values<0.05).

**TFC**: D-lactate administration did not alter the baseline genotype-dependent differences in cue-dependent freezing in TFC in male (E) or female (F) mice; n=4-6 male and 7-15 female mice in each group.

*For male mice*, two-way ANOVA showed a significant effect of genotype, F(1,16)=4.75, P<0.05. Post hoc Bonferroni tests showed that compared with control mice (both treatments), DN-DISC1 mice (both treatments) exhibited less cue-dependent freezing (both p values<0.05).

*For female mice*, two-way ANOVA showed a significant effect of genotype, F(1,23)=5.34, P<0.05. Post hoc Bonferroni tests showed that compared with control mice (both treatments), DN-DISC1 mice (both treatments) exhibited less cue-dependent freezing (both p values<0.05); * denotes p<0.05; data are presented as means ± SEM.

**Supplemental Figure 14. No effects of D-lactate on locomotor activity in EPM or context-dependent freezing in TFC.**

D-lactate administration did not significantly alter locomotor activity in the EPM in male (A) or female (B) mice; n=4-6 male and 6-11 female mice in each group. D-lactate administration did not significantly alter context-dependent freezing in male (C) or female (D) mice; n=4-6 male in each group; n=7-15 female mice in each group; data are presented as means ± SEM.

**Supplemental references**

1. Xia M, Broek JA, Jouroukhin Y, Schoenfelder J, Abazyan S, Jaaro-Peled H*, et al*. Cell Type-Specific Effects of Mutant DISC1: A Proteomics Study. *Molecular neuropsychiatry* 2016; **2**(1)**:** 28-36.

2. Gancarz A, Jouroukhin Y, Saito A, Shevelkin A, Mueller LE, Kamiya A*, et al*. DISC1 signaling in cocaine addiction: Towards molecular mechanisms of co-morbidity. *Neurosci Res* 2016; **105:** 70-74.

3. Ma TM, Abazyan S, Abazyan B, Nomura J, Yang C, Seshadri S*, et al*. Pathogenic disruption of DISC1-serine racemase binding elicits schizophrenia-like behavior via D-serine depletion. *Molecular psychiatry* 2013; **18**(5)**:** 557-567.

4. Gao W, Pu Y, Luo KQ, Chang DC. Temporal relationship between cytochrome c release and mitochondrial swelling during UV-induced apoptosis in living HeLa cells. *J Cell Sci* 2001; **114**(Pt 15)**:** 2855-2862.

5. Cooper O, Seo H, Andrabi S, Guardia-Laguarta C, Graziotto J, Sundberg M*, et al*. Pharmacological rescue of mitochondrial deficits in iPSC-derived neural cells from patients with familial Parkinson's disease. *Science translational medicine* 2012; **4**(141)**:** 141ra190.

6. Abe Y, Sakairi T, Beeson C, Kopp JB. TGF-beta1 stimulates mitochondrial oxidative phosphorylation and generation of reactive oxygen species in cultured mouse podocytes, mediated in part by the mTOR pathway. *American journal of physiology Renal physiology* 2013; **305**(10)**:** F1477-1490.

7. Pletnikov MV, Ayhan Y, Nikolskaia O, Xu Y, Ovanesov MV, Huang H*, et al*. Inducible expression of mutant human DISC1 in mice is associated with brain and behavioral abnormalities reminiscent of schizophrenia. *Molecular psychiatry* 2008; **13**(2)**:** 173-186, 115.

8. Abazyan S, Yang EJ, Abazyan B, Xia M, Yang C, Rojas C*, et al*. Mutant disrupted-in-schizophrenia 1 in astrocytes: focus on glutamate metabolism. *Journal of neuroscience research* 2014; **92**(12)**:** 1659-1668.

9. Terrillion CE, Abazyan B, Yang Z, Crawford J, Shevelkin AV, Jouroukhin Y*, et al*. DISC1 in Astrocytes Influences Adult Neurogenesis and Hippocampus-Dependent Behaviors in Mice. *Neuropsychopharmacology : official publication of the American College of Neuropsychopharmacology* 2017; **42**(11)**:** 2242-2251.

10. Lusk G. *The Elements of the Science of Nutrition, 4th ed*, vol. Reprint, New York: Johnson Reprint Corp., 1976. Academic Press: New York.

11. Pletnikov MV, Ayhan Y, Nikolskaia O, Xu Y, Ovanesov MV, Huang H*, et al*. Inducible expression of mutant human DISC1 in mice is associated with brain and behavioral abnormalities reminiscent of schizophrenia. *Mol Psychiatry* 2008; **13**(2)**:** 173-186, 115.

12. Abazyan B, Dziedzic J, Hua K, Abazyan S, Yang C, Mori S*, et al*. Chronic exposure of mutant DISC1 mice to lead produces sex-dependent abnormalities consistent with schizophrenia and related mental disorders: a gene-environment interaction study. *Schizophr Bull* 2014; **40**(3)**:** 575-584.

13. Abazyan B, Nomura J, Kannan G, Ishizuka K, Tamashiro KL, Nucifora F*, et al*. Prenatal interaction of mutant DISC1 and immune activation produces adult psychopathology. *Biol Psychiatry* 2010; **68**(12)**:** 1172-1181.

14. Ayhan Y, Abazyan B, Nomura J, Kim R, Ladenheim B, Krasnova IN*, et al*. Differential effects of prenatal and postnatal expressions of mutant human DISC1 on neurobehavioral phenotypes in transgenic mice: evidence for neurodevelopmental origin of major psychiatric disorders. *Molecular psychiatry* 2011; **16**(3)**:** 293-306.
